# Dufour effect on unsteady MHD flow past a vertical plate embedded in porous medium with ramped temperature

**DOI:** 10.1038/s41598-022-15603-x

**Published:** 2022-08-03

**Authors:** Subhrajit Sarma, Nazibuddin Ahmed

**Affiliations:** Department of Mathematics, Gauhati University, Guwahati, Assam 781014 India

**Keywords:** Applied mathematics, Software

## Abstract

The present investigation aims to find an exact solution to the problem of a free convective, viscous, radiating, chemically reacting, optically thick, non-gray, and incompressible MHD flow past an exponentially accelerated semi-infinite vertical plate in presence of a transverse magnetic field. The medium of flow is porous. Arbitrary ramped temperature and diffusion thermo effects are also considered. Rosseland approximation method is used to describe the flux that appears in the energy equation. The effects of different parameters on flow and transport characteristics are discussed with the help of suitable graphs. It is noticed that velocity field and concentration field decreases but temperature field increases with an upsurge in Schmidt number. Also, Nusselt number and skin friction rise with increasing chemical reaction parameter but lowers with increasing radiation parameter. Faster consumption of chemical substances decelerates both concentration and velocity but accelerates temperature of the fluid. An interesting outcome outcome of our investigation is that both Dufour effect and arbitrary ramped temperature diminishes fluid velocity.

## Introduction

The branch of physics that deals with the interaction of the magnetic field with electrically conducting fluid are termed Magnetohydrodynamics (MHD). Saltwater, liquid metals, plasmas, electrolytes are some common examples of such fluids. Noted Swiss scientist Hannes Alfven^1^ initiated the field of MHD for which he received the Noble prize in physics in the year 1970. But, due to substantial contributions from other authors like Cowling^2^, Shercliff^3^, Ferraro and Plumpton^4^, Roberts^5^, Crammer and Pai^6^, MHD is at present form. There are several applications of MHD in modern technologies. Geophysical and astrophysical applications of MHD are nicely elaborated by Dormy and Nunez^7^. Dynamo, motor, fusion reactors, dispersion of metals, metallurgy, etc. are some engineering applications of MHD. Aeronautical applications of MHD were studied exclusively by Li et al.^8^. Farrokhi et al.^9^ studied biomedical applications of MHD. Rana et al.^10^ investigated how microbes swim in blood flow of nano- bioconvective Williamson fluid.

Change in fluid temperature and species concentration generates density variation in the fluid mixture. This variation develops buoyancy forces that act on the fluid. The flow produced due to the buoyancy force is termed free convection or natural convection. Manh et al.^11^, Das and Ahmed^12^, Kafoussias^13^, Kumar and Singh^14^, etc. studied the effect of free convection on various MHD problems.

The porous medium contains holes or voids that are filled with solid particles which let the fluid pass through it. The mechanism of porous flow finds its applications in inkjet printing, nuclear waste disposal, electro-chemistry, combustion technology, etc. Dwivedi et al.^15^ studied MHD flow through the vertical channel in a porous medium while Raju et al.^16^ observed the MHD flow through horizontal channel taking viscous dissipation and Joule heating into account. Free convection in the porous media was investigated by Helmy^17^, Raju and Varma^18^, Pattnaik and Biswal^19^, Sinha et al.^20^, Basha and Nagarathna^21^.

Radiation is a form of heat transfer by electromagnetic waves. Many environmental and industrial procedures encounters with radiative convective flows. Flows of this kind take crucial role in space technology and high temperature activities. This influence many authors to perform model research on free convection with thermal radiation in several hydrodynamic and magnetohydrodynamic problems under various physical and geometrical conditions. Mbeldogu et al.^22^, Makinde^23^, Samad and Rahman^24^, Orhan and Ahmet^25^, Prasad et al.^26^, Ahmed and Dutta^27^, Takhar et al.^28^, Seth et al.^29^, Balla and Naikoti^30^, Siviah et al.^31^ are some worth mentioning researchers in this area.

The effect of chemical reaction carries a great practical significance in heat and mass transfer problems. So, many researchers studied applications of chemical reaction in different MHD flow problems. Apelblat^32^ investigated chemical reaction effect in a mass transfer problem with axial diffusion. Mahapatra et al.^33^ examined the effects of chemical reaction in a free convective flow in a porous media surrounded by a vertical surface. Andersson et al.^34^ and Takhar et al.^35^ considered the diffusion of a chemically reactive species from a stretching sheet while Ganesan and Rani^36^ studied the diffusion of chemically reactive species through a vertical cylinder. Muthucumaraswamy and Ganesan^37^, Kandasamy et al.^38^, Raptis and Perdikis^39^, etc. investigated the effects of chemical reaction in various MHD problems. Arifuzzaman et al.^40^ studied chemically reactive and naturally convective high speed MHD flow through an oscillating vertical porous plate.

If two non-reacting and chemically different fluids are allowed to diffuse into each other at the same temperature, the system produces a heat flux. Effect of flux due to composition gradient is defined as Dufour effect or diffusion thermo effect. Renowned Swiss scientist L. Dufour discovered this effect in 1873. This effect is nicely elaborated by Eckert and Drake^41^. Swetha et al.^42^ analyzed Dufour and radiation effects on a free convective flow in a porous medium. Reddy et al.^43^ studied both Soret and Dufour effects of an MHD flow past a moving vertical plate immersed in a porous medium taking Hall current and rotating system into account. Oyekunle and Agunbiade^44^ explored the consequences of the Dufour and Soret effect of MHD flow on an inclined magnetic field. Kumaresan et al.^45^ analytically investigated the Dufour effect on unsteady free convective flow past an accelerated vertical plate. Vijaya Kumar et al.^46^ studied Dufour and radiation effects on a free convective MHD flow past an infinite vertical plate in presence of chemical reaction. Shateyi et al.^47^ studied the effects of Soret, Dufour, Hall current and radiation of a mixed convective flow in a porous medium. Postelnicu^48^ examined the consequences of both Soret and Dufour effects on a vertical surface embedded in a porous medium.

The present investigation aims to analyze the role of the diffusion thermo effect in a free convective, radiative, and chemically reacting fluid in a porous medium with arbitrary ramped temperature. Reviewing the existing literature, we found that no work has been done taking Dufour effect and ramped temperature with arbitrary characteristic time simultaneously in a flow past an exponentially started vertical plate . The governing equations are first converted to non-dimensional partial differential equations using some dimensionless quantities. A closed-form of the Laplace transform technique is adopted to solve the equations. Effects of different flow parameters like Prandtl number, Schmidt number, magnetic parameter, thermal Grashof number, solutal Grashof number, Dufour number, chemical reaction parameter, radiation parameter, porosity parameter, etc. on temperature field, concentration field, velocity field, Nusselt number, Sherwood number, and skin friction are discussed graphically. The obtained results are also verified with previously published work. It is hoped that the present paper will be useful in designing cooling systems, flow meters, MHD generators, etc. In the field of life science, this investigation can be helpful in magnetic drug treatment, devices for cell separation, magnetic endoscopy etc. This paper will also help scientists and researchers in the field of heat and mass transfer.

## Mathematical model of the problem

Equations that govern the convective flow of an electrically conducting, incompressible, viscous, chemically reactive, and radiating fluid in a porous medium in presence of a magnetic field having constant mass diffusivity and thermal diffusivity taking the diffusion- thermo effect into account are.

Continuity equation:

1 $$\overrightarrow {\nabla } \cdot \overrightarrow {q} = 0$$

Magnetic field continuity equation:

2 $$\overrightarrow {\nabla } \cdot \overrightarrow {B} = 0$$

Ohm’s Law:

3 $$\overrightarrow {J} = \sigma \left( {\overrightarrow {E} + \overrightarrow {q} \times \overrightarrow {B} } \right)$$

Momentum equation:

4 $$\rho \left[ {\frac{{\partial \overrightarrow {q} }}{{\partial t^{\prime}}} + \left( {\overrightarrow {q} \cdot \overrightarrow {\nabla } } \right)\overrightarrow {q} } \right] = - \overrightarrow {\nabla } p + \overrightarrow {J} \times \overrightarrow {B} + \rho \overrightarrow {g} + \mu \nabla^{2} \overrightarrow {q} - \frac{{\mu \vec{q}}}{K*}$$

Energy equation:

5 $$\rho C_{p} \left[ {\frac{\partial T}{{\partial t^{\prime}}} + \left( {\overrightarrow {q} \cdot \overrightarrow {\nabla } } \right)T} \right] = \kappa \nabla^{2} T - \overrightarrow {\nabla } \cdot \overrightarrow {{q_{r} }} + \frac{{\rho D_{M} K_{T} }}{{C_{S} }}\nabla^{2} C$$

Species continuity equation:

6 $$\frac{\partial C}{{\partial t^{\prime}}} + \left( {\overrightarrow {q} \cdot \overrightarrow {\nabla } } \right)C = D_{M} \nabla^{2} C + \overline{K}\left( {C_{\infty } - C} \right)$$

Equation of state as per Boussinesq approximation:

7 $$\rho_{\infty } = \rho \left[ {1 + \beta \left( {T - T_{\infty } } \right) + \overline{\beta } \left( {C - C_{\infty } } \right)} \right]$$

The radiation heat flux as per Rosseland approximation is given by

$$\overrightarrow {{q_{r} }} = - \frac{{4\sigma^{*} }}{{3\kappa^{*} }}\overrightarrow {\nabla } T^{4}$$

Now,

$$T^{4} = \left( {T - T_{\infty } + T_{\infty } } \right)^{4} = 4TT_{\infty }^{3} - 3T_{\infty }^{4} ,as\left| {T - T_{\infty } } \right| \ll 1$$

So,

$$\overrightarrow {\nabla } \cdot \overrightarrow {{q_{r} }} = - \frac{{16\sigma^{*} T_{\infty }^{3} }}{{3\kappa^{*} }}\nabla^{2} T$$

Therefore, Energy Eq. (5) reduces to

8 $$\rho C_{p} \left[ {\frac{\partial T}{{\partial t^{\prime}}} + \left( {\overrightarrow {q} \cdot \overrightarrow {\nabla } } \right)T} \right] = \kappa \nabla^{2} T + \frac{{16\sigma^{*} T_{\infty }^{3} }}{{3\kappa^{*} }}\nabla^{2} T + \frac{{\rho D_{M} K_{T} }}{{C_{S} }}\nabla^{2} C$$

We now consider a transient MHD free convection flow of a viscous incompressible electrically conducting fluid through a porous medium past a semi-infinite vertical plate in presence of a uniform magnetic field applied normal to the plate, directed into the fluid region. Initially, the plate and the surrounding fluid were at rest with uniform temperature $T_{\infty }$ and concentration $C_{\infty }$ at all points in the fluid. At time $t^{\prime} > 0$, the plate is exponentially accelerated with velocity $U_{o} e^{{a^{\prime}t^{\prime}}}$. The plate temperature is instantaneously elevated to $T_{\infty } + \left( {T_{w} - T_{\infty } } \right)\frac{{t^{\prime}}}{{t_{0} }}$, for $0 < t^{\prime} \le t_{0}$, and thereafter $T_{w}$ when $t^{\prime} > t_{0}$. The concentration is raised to $C_{w}$ and maintained thereafter.

To idealize the mathematical model, we enforce the following constraints-

I. Except the variation in density in the buoyancy force term, all the fluid properties are constant.
II. Energy dissipation occurring from friction and Joule heating is negligible.
III. Compared to applied magnetic field, induced magnetic field is negligible.
IV. Flow is one- dimensional which is parallel to the plate.
V. The plate is electrically insulating.
VI. Polarization voltage is negligible because no external electric field is applied.

**Figure 1:**
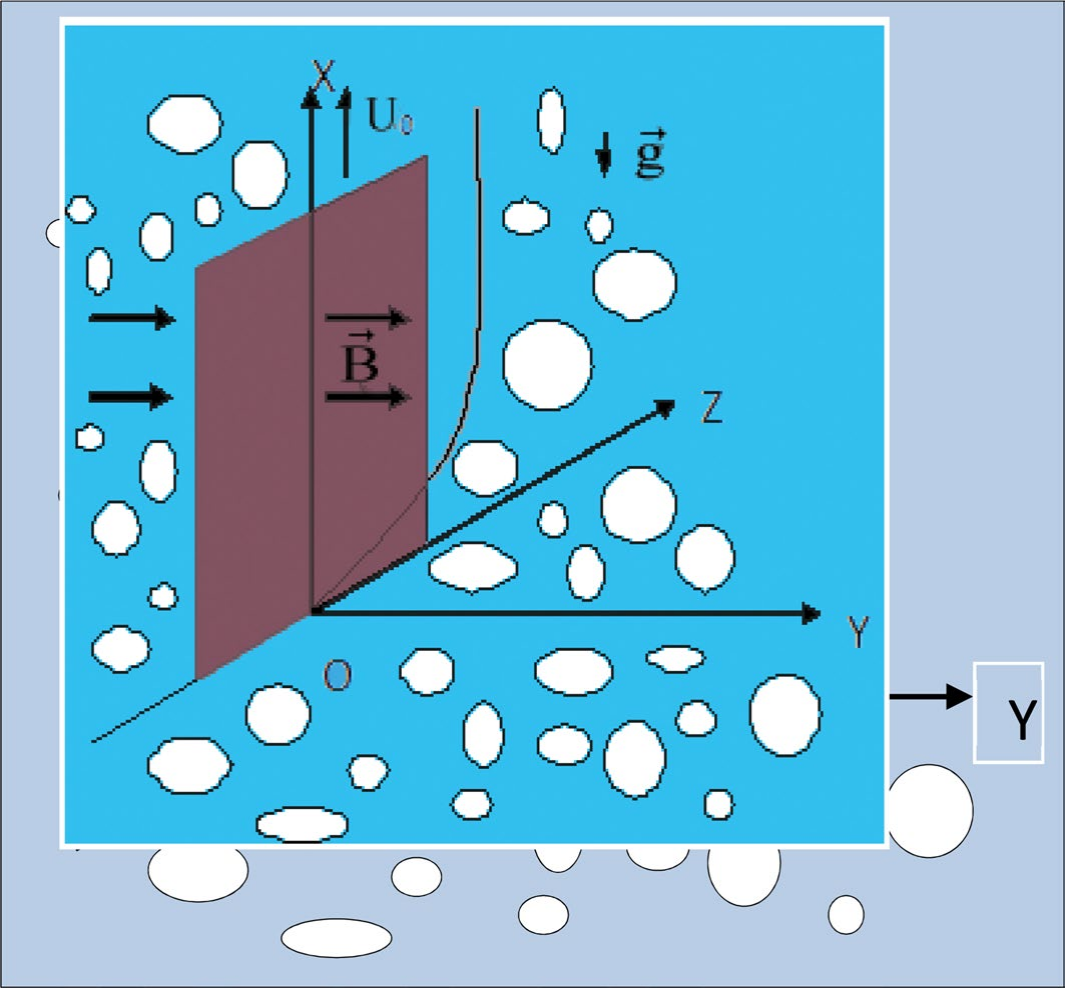
Flow configuration.

We now consider a tri- rectangular Cartesian co-ordinate system $\left( {x^{\prime},y^{\prime},z^{\prime},t^{\prime}} \right)$ with X-axis vertically upwards along the plate, Y-axis normal to the plate directed into the fluid region, and Z-axis along the width of the plate as displayed in Fig. 1. Let $\vec{q} = \left( {u^{\prime},0,0} \right)$ be the fluid velocity and $\vec{B} = \left( {0,B_{0} ,0} \right)$ be the magnetic induction vector at the point $\left( {x^{\prime},y^{\prime},z^{\prime},t^{\prime}} \right)$ in the fluid.

Equation (1) yields,

9 $$\begin{gathered} \frac{{\partial u^{\prime}}}{{\partial x^{\prime}}} = 0 \hfill \\ i.e.,\quad u^{\prime} = u^{\prime}\left( {y^{\prime},t^{\prime}} \right) \hfill \\ \end{gathered}$$

Equation (2) is trivially satisfied by $\vec{B} = \left( {0,B_{0} ,0} \right)$.

Equation (4) reduces to

10 $$\rho \left[ {\frac{{\partial u^{\prime}}}{{\partial t^{\prime}}}\hat{i} + 0} \right] = - \hat{i}\frac{\partial p}{{\partial x^{\prime}}} - \hat{j}\frac{\partial p}{{\partial y^{\prime}}} - \rho g\hat{i} - \sigma B_{0}^{2} u^{\prime}\hat{i} + \mu \frac{{\partial^{2} u^{\prime}}}{{\partial y^{{\prime}{2}} }}\hat{i} - \frac{{\mu u^{\prime}}}{K*}\hat{i}$$

Equation (10) gives

11 $$\rho \frac{{\partial u^{\prime}}}{{\partial t^{\prime}}} = - \frac{\partial p}{{\partial x^{\prime}}} - \rho g - \sigma B_{0}^{2} u^{\prime} + \mu \frac{{\partial^{2} u^{\prime}}}{{\partial y^{{\prime}{2}} }} - \frac{{\mu u^{\prime}}}{K*}$$

And

12 $$0 = - \frac{\partial p}{{\partial y^{\prime}}}$$

Equation (12) shows that pressure near the plate and pressure far away from the plate are the same along the normal to the plate.

For fluid region far away from the plate, Eq. (11) takes the form

13 $$0 = - \frac{\partial p}{{\partial x^{\prime}}} - \rho_{\infty } g$$

Eliminating $\frac{\partial p}{{\partial x^{\prime}}}$ from Eqs. (11) and (13), we get,

14 $$\rho \frac{{\partial u^{\prime}}}{{\partial t^{\prime}}} = \left( {\rho_{\infty } - \rho } \right)g - \sigma B_{0}^{2} u^{\prime} + \mu \frac{{\partial^{2} u^{\prime}}}{{\partial y^{{\prime}{2}} }} - \frac{{\mu u^{\prime}}}{K*}$$

Now, Eq. (7) gives,

15 $$\rho_{\infty } - \rho = \rho \left[ {\beta \left( {T - T_{\infty } } \right) + \overline{\beta }\left( {C - C_{\infty } } \right)} \right]$$

Putting value of Eq. (15) in Eq. (14),

$$\rho \frac{{\partial u^{\prime}}}{{\partial t^{\prime}}} = \rho \left[ {\beta \left( {T - T_{\infty } } \right) + \overline{\beta }\left( {C - C_{\infty } } \right)} \right]g - \sigma B_{0}^{2} u^{\prime} + \mu \frac{{\partial^{2} u^{\prime}}}{{\partial y^{{\prime}{2}} }} - \frac{{\mu u^{\prime}}}{K*}$$

16 $$i.e.,\frac{{\partial u^{\prime}}}{{\partial t^{\prime}}} = g\beta \left( {T - T_{\infty } } \right) + g\overline{\beta }\left( {C - C_{\infty } } \right) - \frac{{\sigma B_{o}^{2} u^{\prime}}}{\rho } + \nu \frac{{\partial^{2} u^{\prime}}}{{\partial y^{{\prime}{2}} }} - \nu \frac{{u^{\prime}}}{K*}$$

Equation (8) yields,

17 $$\rho C_{p} \frac{\partial T}{{\partial t^{\prime}}} = \kappa \frac{{\partial^{2} T}}{{\partial y^{{\prime}{2}} }} + \frac{{16\sigma^{*} T_{\infty }^{3} }}{{3\kappa^{*} }}\frac{{\partial^{2} T}}{{\partial y^{{\prime}{2}} }} + \frac{{\rho D_{M} K_{T} }}{{C_{S} }}\frac{{\partial^{2} C}}{{\partial y^{{\prime}{2}} }}$$

Equation (6) becomes,

18 $$\frac{\partial C}{{\partial t^{\prime}}} = D_{M} \frac{{\partial^{2} C}}{{\partial y^{{\prime}{2}} }} + \overline{K}\left( {C_{\infty } - C} \right)$$

The relevant initial and boundary conditions are:

19 $$\left. \begin{gathered} u^{\prime} = 0,T = T_{\infty } ,C = C_{\infty } :\forall y^{\prime} \ge 0;t^{\prime} \le 0 \hfill \\ u^{\prime} = U_{0} e^{{a^{\prime}t^{\prime}}} ,C = C_{W} :y^{\prime} = 0,t^{\prime} > 0 \hfill \\ T = T_{\infty } + \left( {T_{w} - T_{\infty } } \right)\frac{{t^{\prime}}}{{t_{0} }}:\overline{y} = 0;0 < t^{\prime} \le t_{0} \hfill \\ T = T_{w} :y^{\prime} = 0;t^{\prime} > t_{0} \hfill \\ u^{\prime} \to 0,T \to T_{\infty } ,C \to C_{\infty } :y^{\prime} \to \infty ;t^{\prime} > 0 \hfill \\ \end{gathered} \right\}$$

For the sake of normalization of the mathematical model of the problem, we introduce the following non-dimensional quantities-

$$Du = \frac{{D_{M} K_{T} \left( {C_{w} - C_{\infty } } \right)}}{{C_{S} C_{P} \left( {T_{w} - T_{\infty } } \right)\nu }},N = \frac{{\kappa \kappa^{*} }}{{4\sigma^{*} T_{\infty }^{3} }},u = \frac{{u^{\prime}}}{{U_{0} }},y = \frac{{U_{0} }}{\nu }y^{\prime},t = \frac{{U_{0}^{2} }}{\nu }t^{\prime},Gr = \frac{{\nu g\beta \left( {T_{w} - T_{\infty } } \right)}}{{U_{0}^{3} }},a = a^{\prime}\frac{\nu }{{U_{0}^{2} }},$$

$$Gm = \frac{{\nu g\overline{\beta }\left( {C_{w} - C_{\infty } } \right)}}{{U_{0}^{3} }},\theta = \frac{{T - T_{\infty } }}{{T_{w} - T_{\infty } }},\phi = \frac{{C - C_{\infty } }}{{C_{w} - C_{\infty } }},\Pr = \frac{{\mu C_{p} }}{\kappa },M = \frac{{\sigma B_{0}^{2} \nu }}{{\rho U_{0}^{2} }},Sc = \frac{\nu }{{D_{M} }},\Lambda = 1 + \frac{4}{3N},$$

$$K = \frac{{\nu \overline{K}}}{{U_{0}^{2} }},t_{1} = \frac{{U_{0}^{2} }}{\nu }t_{0} ,M_{1} = M + \frac{1}{K*}$$

The non- dimensional governing equations are

20 $$\frac{\partial u}{{\partial t}} = \frac{{\partial^{2} u}}{{\partial y^{2} }} + Gr\theta + Gm\phi - M_{1} u$$

21 $$\frac{\partial \theta }{{\partial t}} = \frac{\Lambda }{\Pr }\frac{{\partial^{2} \theta }}{{\partial y^{2} }} + Du\frac{{\partial^{2} \phi }}{{\partial y^{2} }}$$

22 $$\frac{\partial \phi }{{\partial t}} = \frac{1}{Sc}\frac{{\partial^{2} \phi }}{{\partial y^{2} }} - K\phi$$

Subject to the initial and boundary conditions

23 $$\left. \begin{gathered} u = 0,\theta = 0,\phi = 0:\forall y \ge 0;t \le 0 \hfill \\ u = e^{at} ,\phi = 1:y = 0,t > 0 \hfill \\ \theta = \frac{t}{{t_{1} }}:y = 0;0 < t \le t_{1} \hfill \\ \theta = 1:y = 0;t > t_{1} \hfill \\ u \to 0,\theta \to 0,\phi \to 0:y \to \infty ;t > 0 \hfill \\ \end{gathered} \right\}$$

## Method of Solution

On taking Laplace transform of the Eqs. (22), (21), and (20) respectively, we get the following equations:

24 $$s\overline{\phi } = \frac{1}{Sc}\frac{{d^{2} \overline{\phi }}}{{dy^{2} }} - K\overline{\phi }$$

25 $$s\overline{\theta } = \frac{\Lambda }{\Pr }\frac{{d^{2} \overline{\theta }}}{{dy^{2} }} + Du\frac{{d^{2} \overline{\phi }}}{{dy^{2} }}$$

26 $$s\overline{u} = \frac{{d^{2} \overline{u}}}{{dy^{2} }} + Gr\overline{\theta } + Gm\overline{\phi } - M_{1} \overline{u}$$

Subject to the initial and boundary conditions:

27 $$\left. \begin{gathered} y = 0:\overline{\theta } = \frac{2}{{s^{2} t_{1} }}\left( {1 - e^{{ - st_{1} }} } \right),\overline{\phi } = \frac{1}{s},\overline{u} = \frac{1}{s - a} \hfill \\ y \to \infty :\overline{\theta } \to 0,\overline{\phi } \to 0,\overline{u} \to 0 \hfill \\ \end{gathered} \right\}$$

Solving equations from Eqs. (24) to (26) subject to the conditions (Eq. 27) and taking inverse Laplace transform of the solutions, the expression for temperature field $\theta$, concentration field $\phi$, and velocity field $u$ are as follows:

28 $$\phi = \psi_{1}$$

29 $$\theta = \left\{ \begin{gathered} \theta_{1,1} + \theta_{1,2} - \theta_{1,3} :\Lambda Sc \ne \Pr \hfill \\ \theta_{2,1} + \theta_{2,2} - \theta_{2,3} :\Lambda Sc = \Pr \hfill \\ \end{gathered} \right.$$

30 $$u = \left\{ \begin{gathered} u_{1,1} + u_{1,2} + u_{1,3} + u_{1,4} + u_{1,5} :\Pr \ne \Lambda ,Sc \ne 1,\Pr \ne \Lambda Sc \hfill \\ u_{2,1} + u_{2,2} + u_{2,3} + u_{2,4} + u_{2,5} :\Pr = \Lambda ,Sc \ne 1 \hfill \\ u_{3,1} + u_{3,2} + u_{3,3} + u_{3,4} + u_{3,5} :\Pr \ne \Lambda ,Sc = 1 \hfill \\ u_{4,1} + u_{4,2} + u_{4,3} + u_{4,4} + u_{4,5} :\Pr = \Lambda ,Sc = 1 \hfill \\ u_{5,1} + u_{5,2} + u_{5,3} + u_{5,4} + u_{5,5} :\Pr \ne \Lambda ,Sc \ne 1,\Pr = \Lambda Sc \hfill \\ \end{gathered} \right.$$

where

$$\psi_{1} = \psi \left( {Sc,K,y,t} \right),a_{1} = \frac{\Pr }{\Lambda },a_{2} = \frac{\Lambda ScK}{{\Lambda Sc - \Pr }},a_{3} = \frac{Du\Pr Sc}{{\Lambda Sc - \Pr }},\theta_{1,1} = \frac{1}{{t_{1} }}\Delta \lambda_{1} ,\lambda_{1} = \lambda \left( {a_{1} ,y,t} \right),$$

$$\theta_{1,2} = a_{3} \left( {A_{1} E_{1} + A_{2} E_{3} } \right),E_{1} = erfc\left( {\frac{{y\sqrt {a_{1} } }}{2\sqrt t }} \right),E_{2} = erfc\left( {\frac{{y\sqrt {a_{1} - a_{2} } }}{2\sqrt t }} \right),E_{3} = e^{{ - a_{2} t}} E_{2} ,A_{1} = \frac{K}{{a_{2} }},$$

$$A_{2} = \frac{{a_{2} - K}}{{a_{2} }},\theta_{1,3} = a_{3} \left( {A_{1} \psi_{1} + A_{2} \psi_{2} } \right),\psi_{2} = \Psi \left( {Sc,K, - a_{2} ,y,t} \right),\theta_{2,1} = \theta_{1,1} ,a_{4} = \frac{Du\Pr }{{\Lambda K}},$$

$$\theta_{2,2} = a_{4} \left( {K\lambda_{1} + P_{1} } \right),P_{1} = P\left( {a_{1} ,y,t} \right),\theta_{2,3} = a_{4} \left( {K\psi_{1} + l_{1} } \right),l_{1} = l\left( {Sc,K,y,t} \right),$$

$$u_{1,1} = u_{1,1,1} + u_{1,1,2} + u_{1,1,3} + u_{1,1,4} + u_{1,1,5} ,u_{1,1,1} = h_{2} ,h_{2} = e^{at} h_{1} ,h_{1} = h\left( {M_{1} + a,y,t} \right),a_{5} = \frac{{M_{1} }}{{a_{1} - 1}},$$

$$a_{6} = \frac{{KSc - M_{1} }}{Sc - 1},a_{7} = \frac{Gr}{{t_{1} \left( {a_{1} - 1} \right)}},u_{1,1,2} = a_{7} \left( {A_{3} \Delta h_{5} + A_{4} \Delta h_{3} + A_{5} \Delta r_{1} } \right),A_{3} = \frac{1}{{a_{5}^{2} }},A_{4} = - A_{3} ,A_{5} = \frac{1}{{a_{5} }},$$

$$h_{3} = h\left( {M_{1} ,y,t} \right),h_{5} = e^{{ - a_{5} t}} h_{4} ,h_{4} = h\left( {M_{1} - a_{5} ,y,t} \right),r_{1} = r\left( {M_{1} ,y,t} \right),a_{8} = \frac{{Gra_{3} }}{{a_{1} - 1}},$$

$$\begin{gathered} u_{1,1,3} = a_{8} \left( {A_{6} h_{7} + A_{7} h_{5} + A_{8} h_{3} } \right),A_{6} = \frac{{a_{2} - K}}{{a_{2} \left( {a_{5} - a_{2} } \right)}},A_{7} = \frac{{a_{5} - K}}{{a_{5} \left( {a_{2} - a_{5} } \right)}},A_{8} = \frac{K}{{a_{2} a_{5} }},h_{7} = e^{{ - a_{2} t}} h_{6} , \hfill \\ h_{6} = h\left( {M_{1} - a_{2} ,y,t} \right),a_{9} = \frac{{Gra_{3} }}{Sc - 1},u_{1,1,4} = a_{9} \left( {A_{9} h_{7} + A_{10} h_{9} + A_{11} h_{3} } \right),h_{9} = e^{{ - a_{6} t}} h_{8} ,h_{8} = h\left( {M_{1} - a_{6} ,y,t} \right), \hfill \\ \end{gathered}$$

$$A_{9} = \frac{{a_{2} - K}}{{a_{2} \left( {a_{6} - a_{2} } \right)}},A_{10} = \frac{{a_{6} - K}}{{a_{6} \left( {a_{2} - a_{6} } \right)}},A_{11} = \frac{K}{{a_{2} a_{6} }},a_{10} = \frac{Gm}{{Sc - 1}},u_{1,1,5} = a_{10} \left( {A_{12} h_{9} + A_{13} h_{3} } \right),A_{12} = - \frac{1}{{a_{6} }},$$

$$A_{13} = - A_{12} ,u_{1,2} = - a_{7} \left( {A_{3} \Delta E_{5} + A_{4} \Delta E_{1} + A_{5} \Delta \lambda_{1} } \right),E_{5} = e^{{ - a_{5} t}} E_{4} ,E_{4} = erfc\left( {\frac{{y\sqrt {a_{1} - a_{5} } }}{2\sqrt t }} \right),$$

$$u_{1,3} = - a_{8} \left( {A_{6} E_{3} + A_{7} E_{5} + A_{8} E_{1} } \right),u_{1,4} = - a_{9} \left( {A_{9} \psi_{2} + A_{10} \psi_{3} + A_{11} \psi_{1} } \right),\psi_{3} = \Psi \left( {Sc,K, - a_{6} ,y,t} \right),$$

$$u_{1,5} = - a_{10} \left( {A_{12} \psi_{3} + A_{13} \psi_{1} } \right),u_{2,1} = u_{2,1,1} + u_{2,1,2} + u_{2,1,3} + u_{2,1,4} + u_{2,1,5} ,u_{2,1,1} = u_{1,1,1} ,a_{11} = \frac{Gr}{{M_{1} t_{1} }},$$

$$u_{2,1,2} = a_{11} \Delta r_{1} ,a_{12} = \frac{{Gra_{3} }}{{M_{1} }},u_{2,1,3} = a_{12} \left( {A_{14} h_{7} + A_{15} h_{3} } \right),A_{14} = - \frac{K}{{a_{2} }},A_{15} = - A_{14} ,u_{2,1,4} = u_{1,1,4} ,u_{2,1,5} = u_{1,1,5} ,$$

$$u_{2,2} = - a_{11} \Delta \lambda_{2} ,\lambda_{2} = \lambda \left( {1,y,t} \right),u_{2,3} = - a_{12} \left( {A_{14} E_{8} + A_{15} E_{6} } \right),E_{6} = erfc\left( {\frac{y}{2\sqrt t }} \right),E_{8} = e^{{ - a_{2} t}} E_{7} ,$$

$$E_{7} = erfc\left( {\frac{{y\sqrt {1 - a_{2} } }}{2\sqrt t }} \right),u_{2,4} = u_{1,4} ,u_{2,5} = u_{1,5} ,u_{3,1} = u_{3,1,1} + u_{3,1,2} + u_{3,1,3} + u_{3,1,4} + u_{3,1,5} ,u_{3,1,1} = u_{1,1,1} ,$$

$$u_{3,1,2} = u_{1,1,2} ,u_{3,1,3} = u_{1,1,3} ,u_{3,1,4} = a_{13} \left( {A_{14} h_{7} + A_{15} h_{3} } \right),a_{13} = \frac{{Gra_{3} }}{{K - M_{1} }},u_{3,1,5} = a_{14} h_{3} ,a_{14} = \frac{Gm}{{K - M_{1} }},$$

$$u_{3,2} = u_{1,2} ,u_{3,3} = u_{1,3} ,u_{3,4} = - a_{13} \left( {A_{14} h_{11} + A_{15} h_{12} } \right),h_{10} = h\left( {K - a_{2} ,y,t} \right),h_{11} = e^{{ - a_{2} t}} h_{10} ,h_{12} = h\left( {K,y,t} \right),$$

$$u_{3,5} = - a_{13} \psi_{1} ,u_{4,1} = u_{4,1,1} + u_{4,1,2} + u_{4,1,3} + u_{4,1,4} + u_{4,1,5} ,u_{4,1,1} = u_{1,1,1} ,u_{4,1,2} = u_{2,1,2} ,a_{15} = \frac{GrDu}{{KM_{1} }},$$

$$u_{4,1,3} = a_{15} \left( {Kh_{3} + q_{1} } \right),q_{1} = q\left( {M_{1} ,y,t} \right),u_{4,1,4} = a_{16} \left( {Kh_{3} + q_{1} } \right),u_{4,1,5} = a_{17} h_{3} ,a_{17} = \frac{Gm}{{K - M_{1} }},u_{4,2} = u_{2,2} ,$$

$$u_{4,3} = - a_{15} E_{6} ,E_{6} = erfc\left( {\frac{y}{2\sqrt t }} \right),u_{4,4} = - a_{16} \left( {Kh_{12} + q_{2} } \right),h_{12} = h\left( {K,y,t} \right),q_{2} = q\left( {K,y,t} \right),$$

$$u_{4,5} = - a_{17} h_{10} ,u_{5,1} = u_{5,1,1} + u_{5,1,2} + u_{5,1,3} + u_{5,1,4} + u_{5,1,5} ,u_{5,1,1} = u_{1,1,1} ,u_{5,1,2} = u_{1,1,2} ,$$

$$u_{5,1,3} = a_{18} \left( {A_{16} h_{5} + A_{17} h_{3} } \right),a_{18} = \frac{GrDuSc}{{\left( {a_{1} - 1} \right)K}},A_{16} = \frac{{a_{5} - K}}{{a_{5} }},A_{17} = \frac{K}{{a_{5} }},u_{5,1,4} = a_{18} \left( {A_{18} h_{3} + A_{19} h_{9} } \right),$$

$$A_{18} = \frac{K}{{a_{6} }},A_{19} = \frac{{a_{6} - K}}{{a_{6} }},u_{5,1,5} = u_{1,1,5} ,u_{5,2} = u_{1,2} ,u_{5,3} = - a_{18} \left( {A_{16} \psi_{4} + A_{17} \psi_{1} } \right),\psi_{4} = \Psi \left( {Sc,K, - a_{5} ,y,t} \right),$$

$$u_{5,4} = - a_{18} \left( {A_{18} \psi_{1} + A_{19} \psi_{3} } \right),u_{5,5} = u_{1,5}$$

## Nusselt number

The heat flux $q^{*}$ at the plate $y^{\prime} = 0$ is obtained by Fourier’s law of conduction is given by

31 $$q^{*} = \left. { - \kappa_{0}^{*} \frac{\partial T}{{\partial y^{\prime}}}} \right]_{{y^{\prime} = 0}}$$

where $\kappa_{0}^{*} = \kappa + \frac{{16\sigma^{*} T_{\infty }^{3} }}{{3\kappa^{*} }}$ is the modified thermal conductivity.

Equation (31) yields

32 $$Nu = - \left. {\frac{\partial \theta }{{\partial y}}} \right]_{y = 0}$$

where $Nu = \frac{{q^{*} \nu }}{{\kappa_{0}^{*} U_{0} \left( {T_{w} - T_{\infty } } \right)}}$ $= \frac{{3Nq^{*} \nu }}{{\kappa \left( {4 + 3N} \right)\left( {T_{w} - T_{\infty } } \right)U_{0} }}$ is called the Nusselt number which is concerned with the rate of heat transfer at the plate.

Equation (32) gives,

33 $$Nu = - \left\{ \begin{gathered} Nu_{1,1} + Nu_{1,2} - Nu_{1,3} :\Lambda Sc \ne \Pr \hfill \\ Nu_{2,1} + Nu_{2,2} - Nu_{2,3} :\Lambda Sc = \Pr \hfill \\ \end{gathered} \right.$$

where

$$Nu_{1,1} = \frac{1}{{t_{1} }}\Delta \nu_{1} ,\nu_{1} = \nu \left( {a_{1} ,t} \right),Nu_{1,2} = a_{3} \left( {A_{1} \alpha_{1} + A_{2} \alpha_{3} } \right),\alpha_{1} = \alpha \left( {\frac{{\sqrt {a_{1} } }}{2\sqrt t }} \right),\alpha_{2} = \alpha \left( {\frac{{\sqrt {a_{1} - a_{2} } }}{2\sqrt t }} \right),$$

$$\alpha_{3} = e^{{ - a_{2} t}} \alpha_{2} ,Nu_{1,3} = a_{3} \left( {A_{1} \Omega_{1} + A_{2} Z_{1} } \right),\Omega_{1} = \Omega \left( {Sc,K,t} \right),Z_{1} = Z\left( {Sc,K, - a_{2} ,t} \right),Nu_{2,1} = Nu_{1,1} ,$$

$$Nu_{2,2} = a_{4} \left( {K\nu_{1} + I_{1} } \right),I_{1} = I\left( {a_{1} ,t} \right),Nu_{2,3} = a_{4} \left( {K\Omega_{1} + T_{1} } \right),T_{1} = T\left( {Sc,K,t} \right)$$

## Sherwood number

The mass flux $M_{w}$ at the plate $y^{\prime} = 0$ is specified by Fick’s law of diffusion is given by

34 $$M_{w} = - \left. {D_{M} \frac{\partial C}{{\partial y^{\prime}}}} \right]_{{y^{\prime} = 0}}$$

Equation (34) gives

35 $$Sh = - \left. {\frac{\partial \phi }{{\partial y}}} \right]_{y = 0}$$

In Eq. (35), $Sh = \frac{{M_{w} \nu }}{{D_{M} U_{0} \left( {C_{w} - C_{\infty } } \right)}}$ is called the Sherwood number which is associated with the rate of mass transfer at the plate.

Equation (35) yields

36 $$Sh = - \Omega_{1}$$

## Skin friction

The viscous drag at the plate $y^{\prime} = 0$ is determined by Newton’s law of viscosity is given by

37 $$\overline{\tau } = - \left. {\mu \frac{\partial u}{{\partial y^{\prime}}}} \right]_{{y^{\prime} = 0}}$$

Equation (37) gives

38 $$\tau = - \left. {\frac{\partial u}{{\partial y}}} \right]_{y = 0}$$

In Eq. (38), $\tau = \frac{{\overline{\tau }\nu }}{{\mu U_{0}^{2} }}$ is called the skin friction or coefficient of friction which is associated with the rate of momentum transfer at the plate.

Equation (38) yields,

39 $$\tau = - \left\{ \begin{gathered} \tau_{1,1} + \tau_{1,2} + \tau_{1,3} + \tau_{1,4} + \tau_{1,5} :\Pr \ne \Lambda ,Sc \ne 1,\Pr \ne \Lambda Sc \hfill \\ \tau_{2,1} + \tau_{2,2} + \tau_{2,3} + \tau_{2,4} + \tau_{2,5} :\Pr = \Lambda ,Sc \ne 1 \hfill \\ \tau_{3,1} + \tau_{3,2} + \tau_{3,3} + \tau_{3,4} + \tau_{3,5} :\Pr \ne \Lambda ,Sc = 1 \hfill \\ \tau_{4,1} + \tau_{4,2} + \tau_{4,3} + \tau_{4,4} + \tau_{4,5} :\Pr = \Lambda ,Sc = 1 \hfill \\ \tau_{5,1} + \tau_{5,2} + \tau_{5,3} + \tau_{5,4} + \tau_{5,5} :\Pr \ne \Lambda ,Sc \ne 1,\Pr = \Lambda Sc \hfill \\ \end{gathered} \right.$$

where

$$\tau_{1,1} = \tau_{1,1,1} + \tau_{1,1,2} + \tau_{1,1,3} + \tau_{1,1,4} ,\tau_{1,1,1} = N_{2} ,N_{2} = e^{at} N_{1} ,N_{1} = N\left( {M_{1} + a,t} \right),$$

$$\tau_{1,1,2} = a_{7} \left( {A_{3} \Delta N_{5} + A_{4} \Delta N_{3} + A_{5} \Delta O_{1} } \right),N_{3} = N\left( {M_{1} ,t} \right),N_{4} = N\left( {M_{1} - a_{5} ,t} \right),N_{5} = e^{{ - a_{5} t}} N_{4} ,$$

$$O_{1} = O\left( {M_{1} ,t} \right),\tau_{1,1,3} = a_{8} \left( {A_{6} N_{7} + A_{7} N_{5} + A_{8} N_{3} } \right),N_{7} = e^{{ - a_{2} t}} N_{6} ,N_{6} = N\left( {M_{1} - a_{2} ,t} \right),$$

$$\tau_{1,1,4} = a_{9} \left( {A_{9} N_{7} + A_{10} N_{9} + A_{11} N_{3} } \right),N_{9} = e^{{ - a_{6} t}} N_{8} ,N_{8} = N\left( {M_{1} - a_{6} ,t} \right),\tau_{1,1,5} = a_{10} \left( {A_{12} N_{9} + A_{13} N_{3} } \right),$$

$$\begin{gathered} \tau_{1,2} = - a_{7} \left( {A_{3} \Delta \alpha_{5} + A_{4} \Delta \alpha_{1} + A_{5} \Delta \nu_{1} } \right),\alpha_{5} = e^{{ - a_{5} t}} \alpha_{4} ,\alpha_{4} = \alpha \left( {\frac{{\sqrt {a_{1} - a_{5} } }}{2\sqrt t }} \right), \hfill \\ \tau_{1,3} = - a_{8} \left( {A_{6} \alpha_{3} + A_{7} \alpha_{5} + A_{8} \alpha_{1} } \right),\tau_{1,4} = - a_{9} \left( {A_{9} Z_{1} + A_{10} Z_{2} + A_{11} \Omega_{1} } \right),Z_{2} = Z\left( {Sc,K, - a_{6} ,t} \right), \hfill \\ \end{gathered}$$

$$\tau_{1,5} = - a_{10} \left( {A_{12} Z_{2} + A_{3} \Omega_{1} } \right),\tau_{2,1} = \tau_{2,1,1} + \tau_{2,1,2} + \tau_{2,1,3} + \tau_{2,1,4} + \tau_{2,1,5} ,\tau_{2,1,1} = \tau_{1,1,1} ,\tau_{2,1,2} = a_{11} \Delta O_{1} ,$$

$$\tau_{2,1,3} = a_{12} \left( {A_{14} N_{7} + A_{15} N_{3} } \right),\tau_{2,1,4} = \tau_{1,1,4} ,\tau_{2,1,5} = \tau_{1,1,5} ,\tau_{2,2} = - a_{11} \Delta \nu_{2} ,\nu_{2} = \nu \left( {1,t} \right),$$

$$\tau_{2,3} = - a_{12} \left( {A_{14} \alpha_{8} + A_{15} \alpha_{6} } \right),\alpha_{6} = \alpha \left( {\frac{1}{2\sqrt t }} \right),\alpha_{8} = e^{{ - a_{2} t}} \alpha_{7} ,\alpha_{7} = \alpha \left( {\frac{{\sqrt {1 - a_{2} } }}{2\sqrt t }} \right),\tau_{2,4} = \tau_{1,4} ,\tau_{2,5} = \tau_{1,5} ,$$

$$\tau_{3,1} = \tau_{3,1,1} + \tau_{3,1,2} + \tau_{3,1,3} + \tau_{3,1,4} + \tau_{3,1,5} ,\tau_{3,1,1} = \tau_{1,1,1} ,\tau_{3,1,2} = \tau_{1,1,2} ,\tau_{3,1,3} = \tau_{1,1,3} ,$$

$$\tau_{3,1,4} = a_{13} \left( {A_{14} N_{7} + A_{15} N_{3} } \right),\tau_{3,1,5} = a_{14} N_{3} ,\tau_{3,2} = \tau_{1,2} ,\tau_{3,3} = \tau_{1,3} ,\tau_{3,4} = - a_{13} \left( {A_{14} N_{11} + A_{15} N_{12} } \right),$$

$$\begin{gathered} \tau_{3,5} = - a_{13} \Omega_{1} ,N_{10} = N\left( {K - a_{2} ,t} \right),N_{11} = e^{{ - a_{2} t}} N_{10} ,N_{12} = N\left( {K,t} \right), \hfill \\ \tau_{4,1} = \tau_{4,1,1} + \tau_{4,1,2} + \tau_{4,1,3} + \tau_{4,1,4} + \tau_{4,1,5} ,\tau_{4,1,1} = \tau_{1,1,1} ,\tau_{4,1,2} = \tau_{2,1,2} ,\tau_{4,1,3} = a_{15} \left( {KN_{3} + Y_{1} } \right),Y_{1} = Y\left( {M_{1} ,t} \right), \hfill \\ \end{gathered}$$

$$\tau_{4,1,4} = a_{16} \left( {KN_{3} + Y_{1} } \right),\tau_{4,1,5} = a_{17} N_{3} ,\tau_{4,2} = \tau_{2,2} ,\tau_{4,3} = - a_{15} \alpha_{6} ,\alpha_{6} = \alpha \left( {\frac{1}{2\sqrt t }} \right),\tau_{4,4} = - a_{16} \left( {KN_{12} + Y_{2} } \right),$$

$$Y_{2} = Y\left( {K,t} \right),\tau_{4,5} = - a_{17} N_{12} ,\tau_{5,1} = \tau_{5,1,1} + \tau_{5,1,2} + \tau_{5,1,3} + \tau_{5,1,4} + \tau_{5,1,5} ,\tau_{5,1,1} = \tau_{1,1,1} ,\tau_{5,1,2} = \tau_{1,1,2} ,$$

$$\tau_{5,1,3} = a_{18} \left( {A_{16} N_{5} + A_{17} N_{3} } \right),\tau_{5,1,4} = a_{18} \left( {A_{18} N_{3} + A_{19} N_{9} } \right),\tau_{5,1,5} = \tau_{1,1,5} ,\tau_{5,2} = \tau_{1,2} ,$$

$$\tau_{5,3} = - a_{18} \left( {A_{16} Z_{3} + A_{17} \Omega_{1} } \right),Z_{3} = Z\left( {Sc,K, - a_{5} ,t} \right),\tau_{5,4} = - a_{18} \left( {A_{18} \Omega_{1} + A_{19} Z_{2} } \right),\tau_{5,5} = \tau_{1,5}$$

## Result and discussion

The effects of various flow parameters associated with the flow and transport properties are examined by assigning some specific values. The results are demonstrated from Figs. 2, 3, 4, 5, 6, 7, 8, 9, 10, 11, 12, 13, 14, 15, 16, 17, 18, 19, 20, 21, 22, 23, 24, 25, 26, 27, 28, 29, 30, 31, 32, 33, 34 and 35.

**Figure 2:**
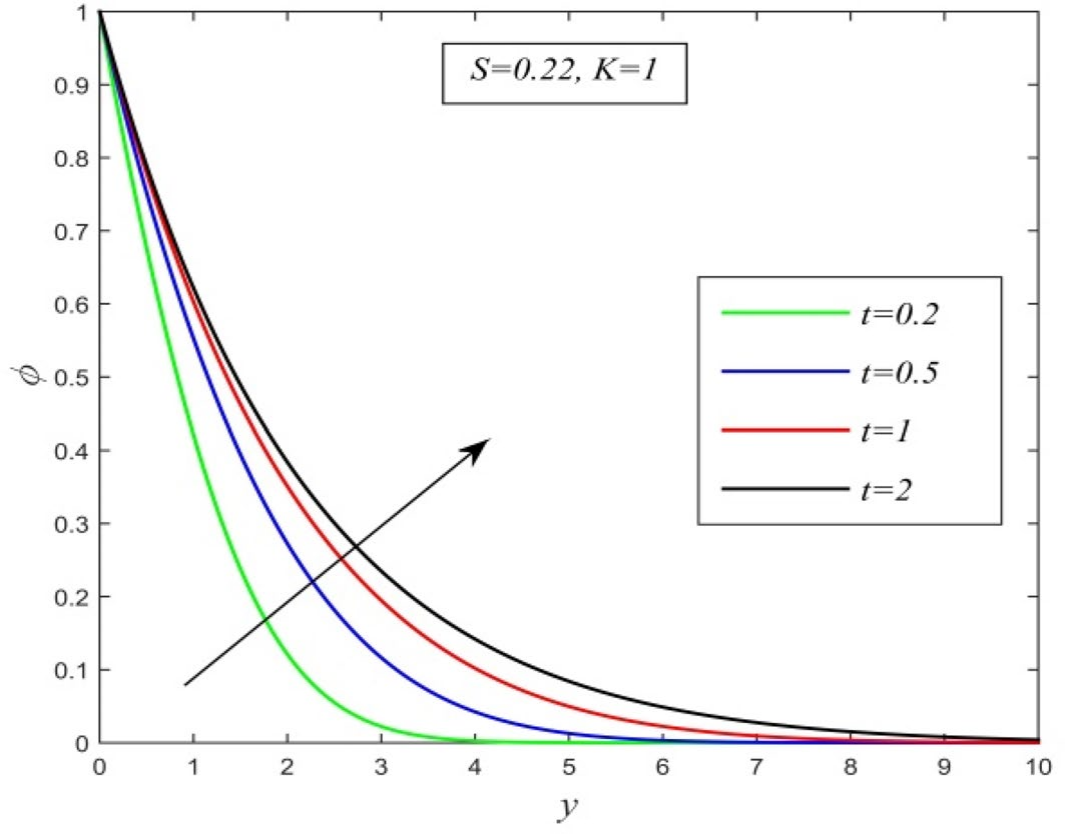
versus $y$ for different *t* and *Sc* = 0.22, *K* = 1.

**Figure 3:**
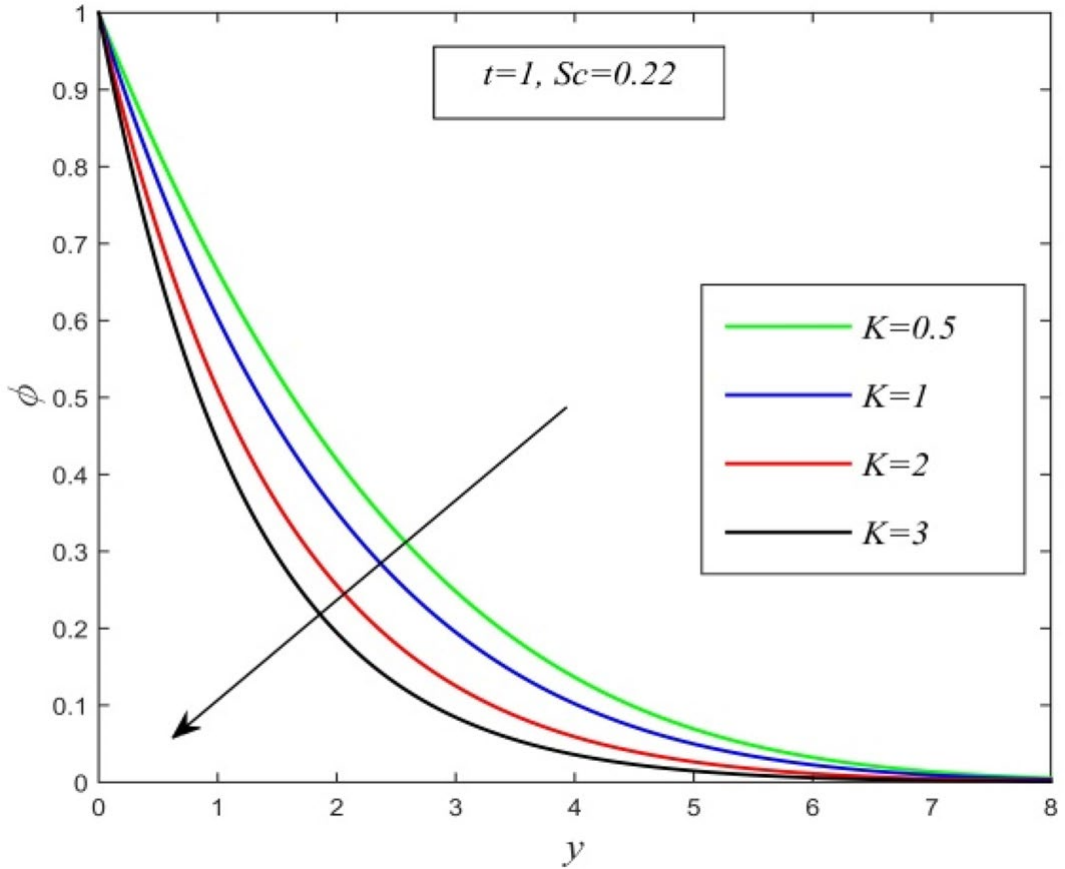
$\phi$ versus $y$ for different *K* and *t* = 1, *Sc* = 0.22.

**Figure 4:**
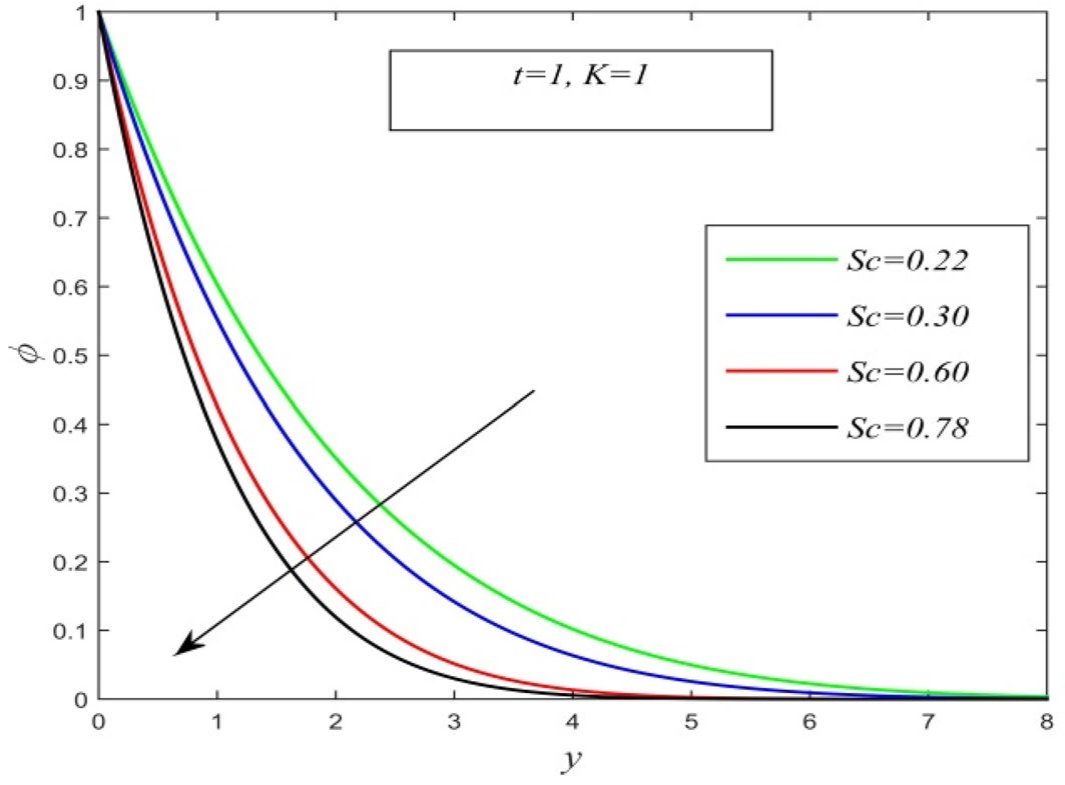
$\phi$ versus $y$ for different *Sc* and *t* = 1, *K* = 0.22.

**Figure 5:**
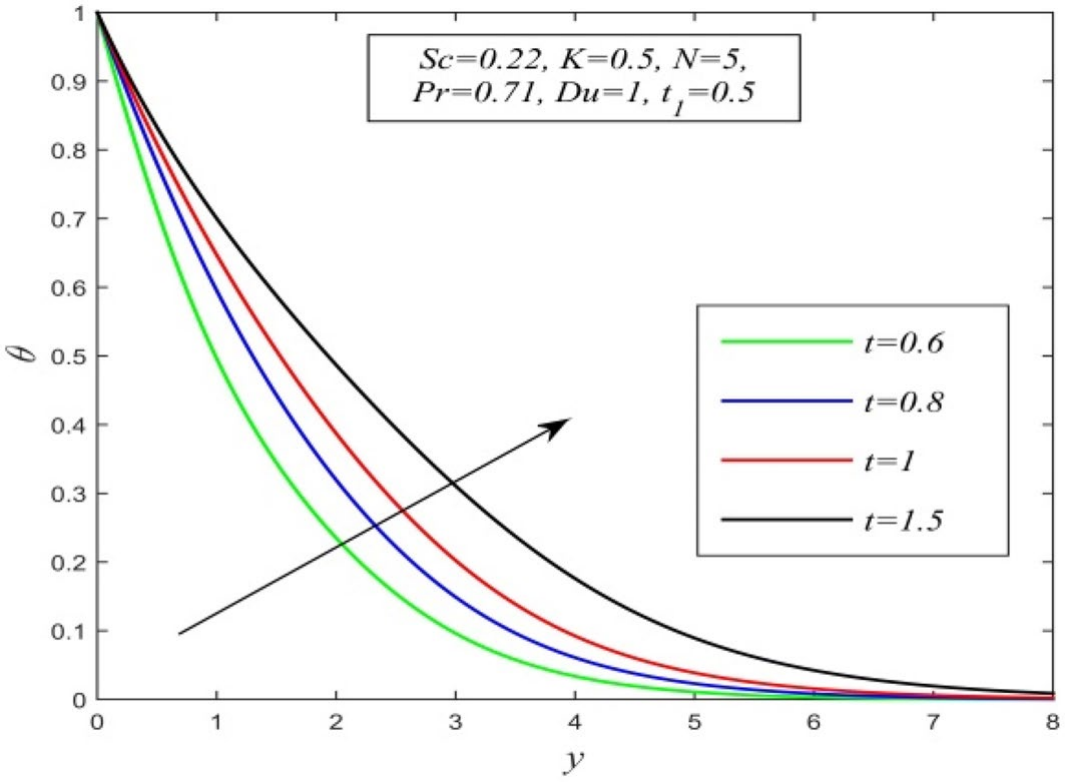
$\theta$ versus *y* for different *t* and *Sc* = 0.22, *K* = 0.5, *N* = 5, *Pr* = 0.71, *Du* = 1, $t_{1}$ = 0.5.

**Figure 6:**
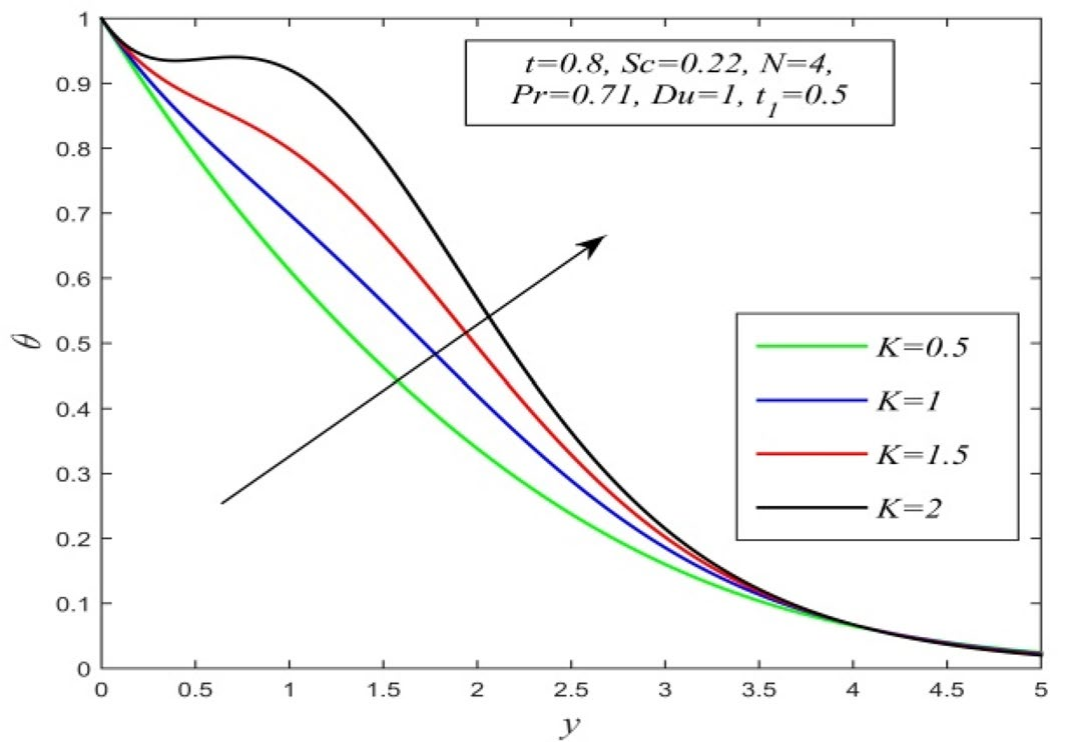
$\theta$ versus *y* for different *K* and *t* = 0.8, *Sc* = 0.22, *N* = 4, *Pr* = 0.71, *Du* = 1, $t_{1}$ = 0.5.

**Figure 7:**
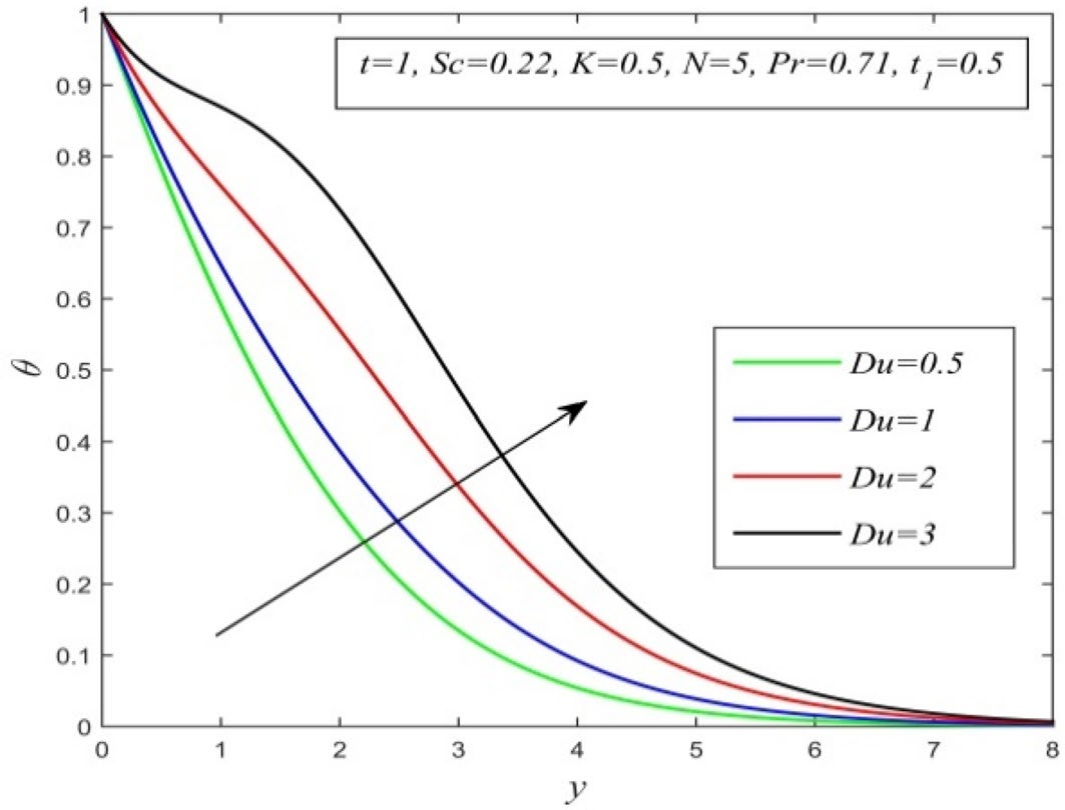
$\theta$ versus *y* for different *Du* and *t* = 1, *Sc* = 0.22, *K* = 0.5, *N* = 5, *Pr* = 0.71, $t_{1}$ = 0.5.

**Figure 8:**
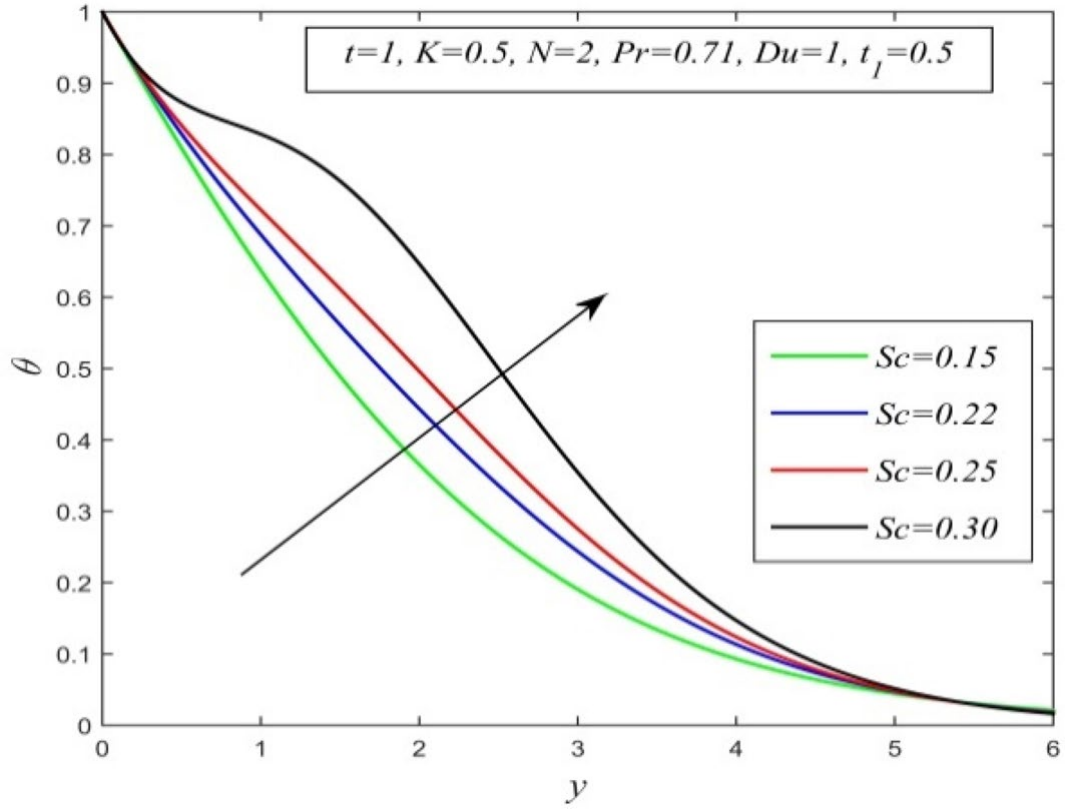
$\theta$ versus *y* for different *Sc* and *t* = 1, *K* = 0.5, *N* = 2, *Pr* = 0.71, *Du* = 1, $t_{1}$ = 0.5.

**Figure 9:**
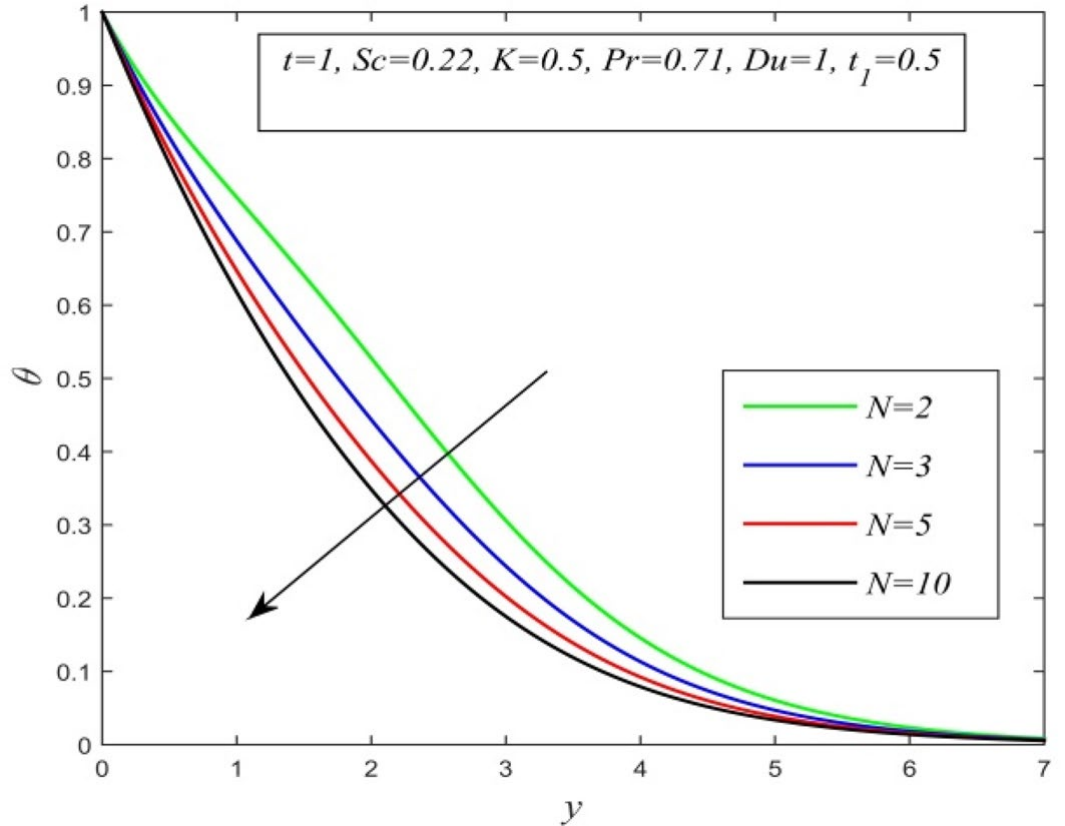
$\theta$ versus *y* for different *N* and *t* = 1, *Sc* = 0.22, *K* = 0.5, *Pr* = 0.71, *Du* = 1, $t_{1}$ = 0.5.

**Figure 10:**
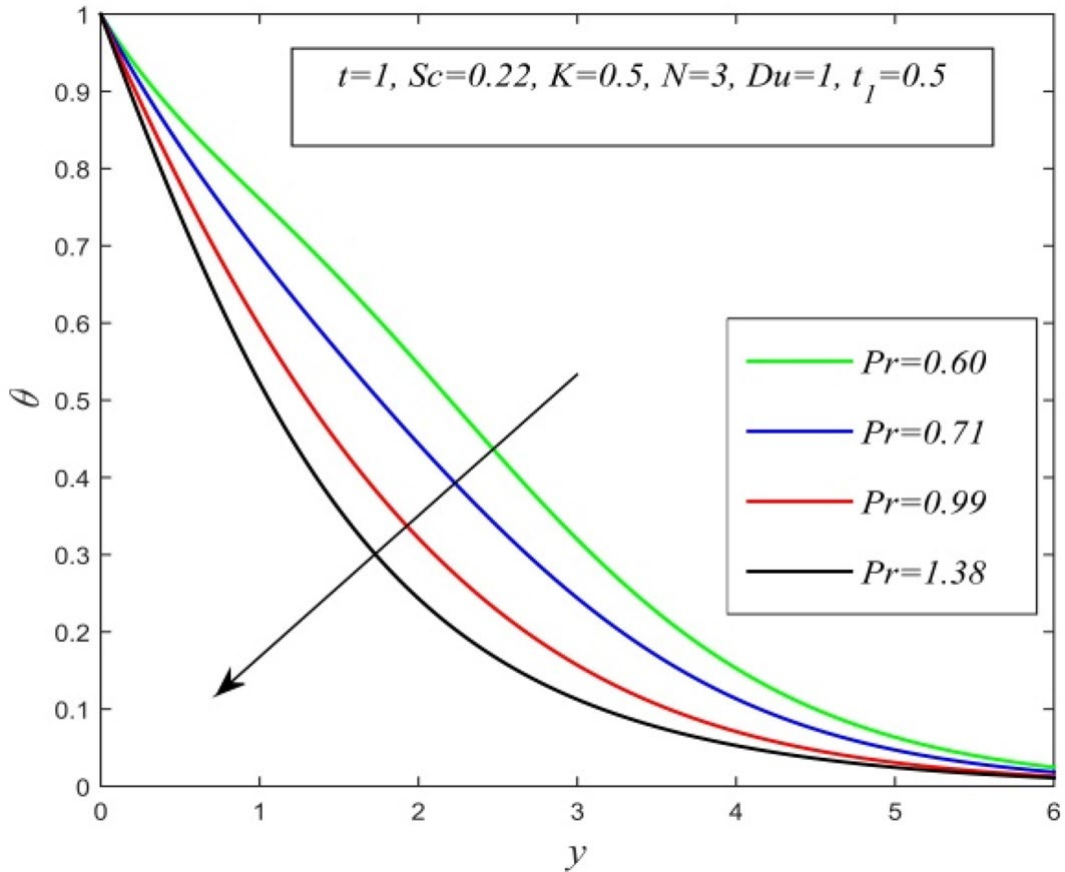
$\theta$ versus *y* for different *Pr* and *t* = 1, *Sc* = 0.22, *K* = 0.5, *N* = 3, *Du* = 1, $t_{1}$ = 0.5.

**Figure 11:**
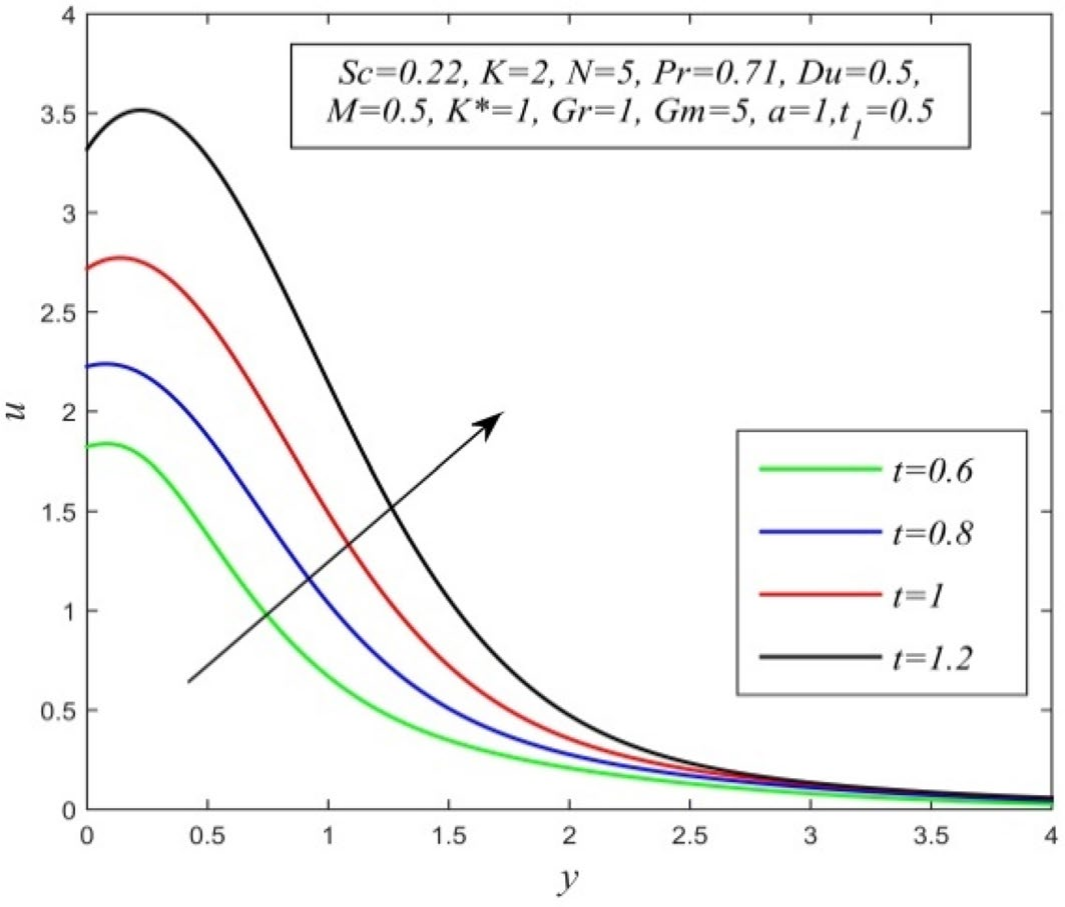
*u* versus *y* for different *t* and *Sc* = 0.22, *K* = 2, *N* = 5, *Pr* = 0.71, *Du* = 0.5, *M* = 0.5, K* = 1, *Gr* = 1, *Gm* = 10, *a* = 1, $t_{1}$ = 0.5.

**Figure 12:**
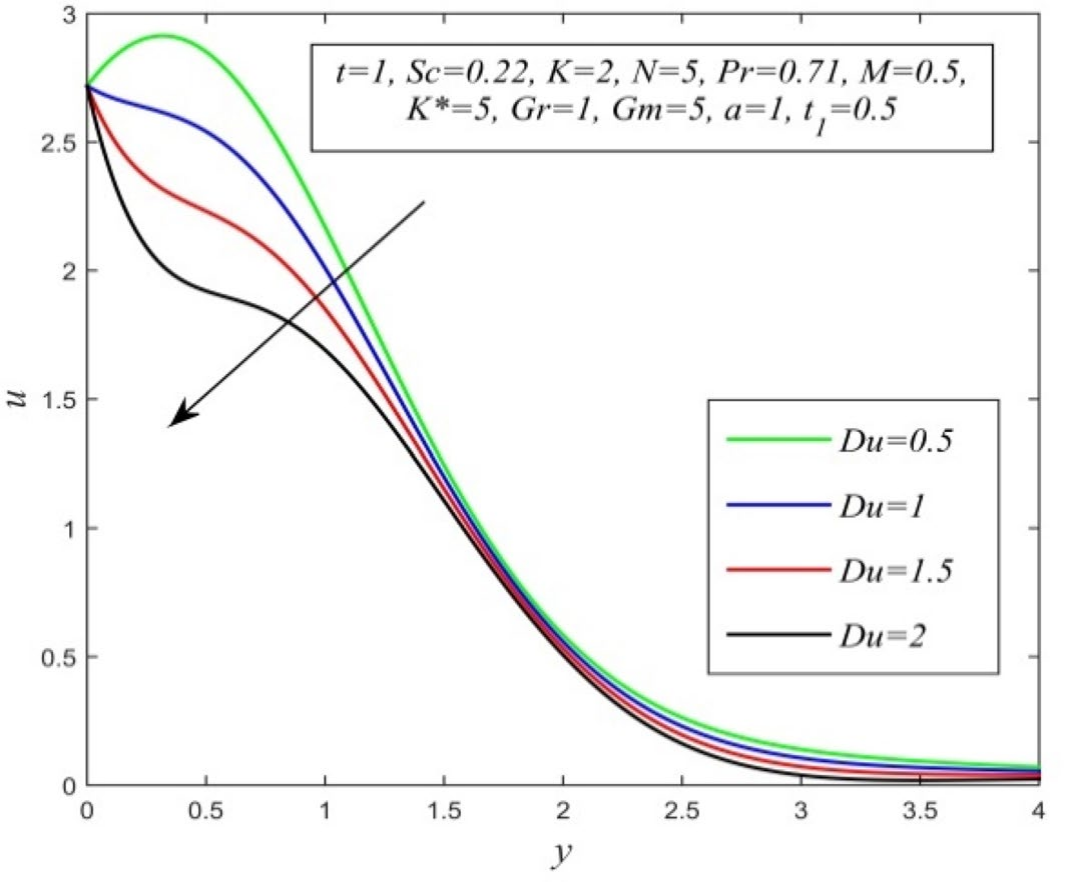
*u* versus *y* for different *Du* and *t* = 1, *Sc* = 0.22, *K* = 2, *N* = 5, *Pr* = 0.71, *M* = 0.5, K* = 1, *Gr* = 1, *Gm* = 5, *a* = 1, $t_{1}$ = 0.5.

**Figure 13:**
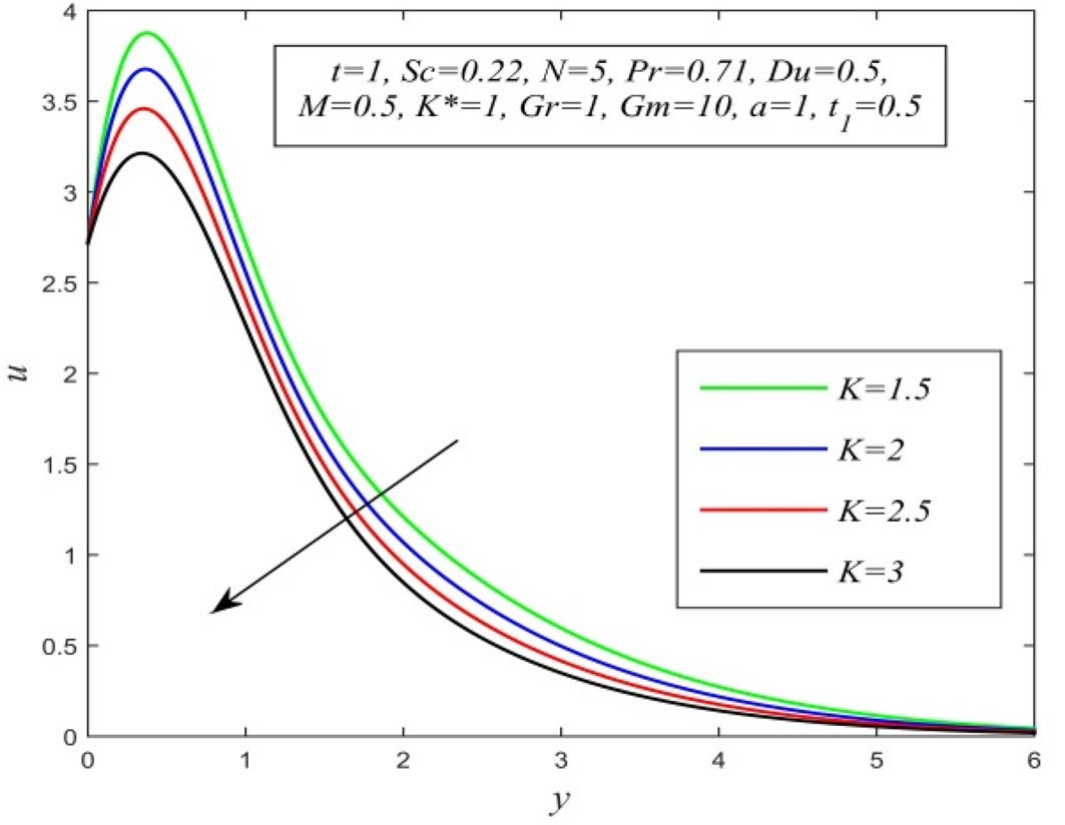
*u* versus *y* for different *K* and *t* = 1, *Sc* = 0.22, *N* = 5, *Pr* = 0.71, *Du* = 0.5, *M* = 0.5, K* = 1, *Gr* = 1, *Gm* = 10, *a* = 1, $t_{1}$ = 0.5.

**Figure 14:**
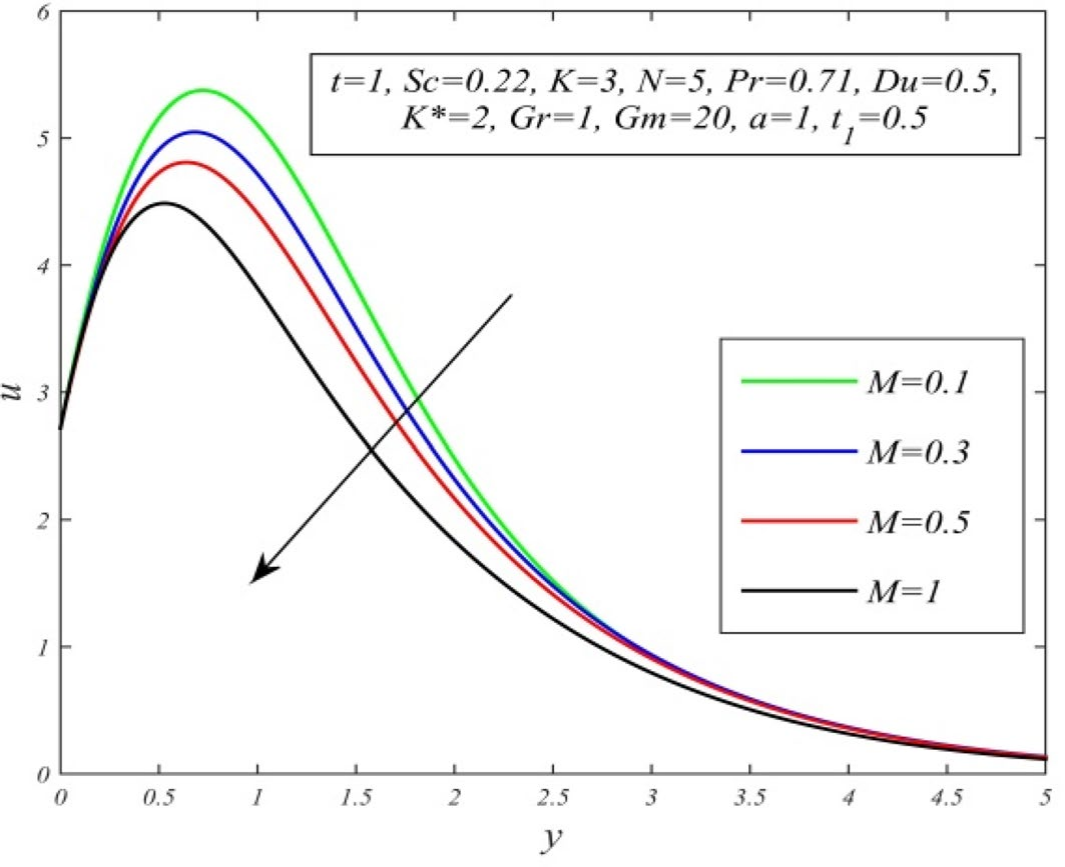
*u* versus *y* for different *M* and *t* = 1, *Sc* = 0.22, *K* = 3, *N* = 5, *Pr* = 0.71, *Du* = 0.5, K* = 2, *Gr* = 1, *Gm* = 20, *a* = 1, $t_{1}$ = 0.5.

**Figure 15:**
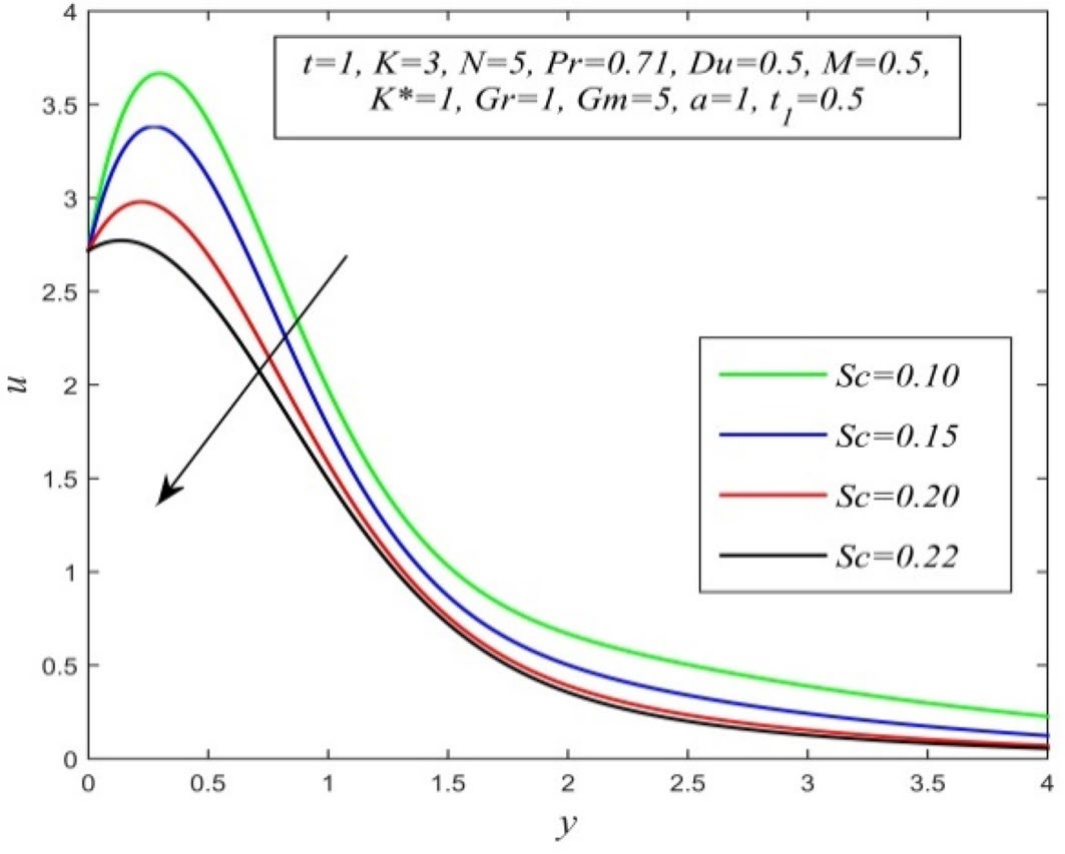
*u* versus *y* for different *Sc* and *t* = 1, *K* = 3, *N* = 5, *Pr* = 0.71, *Du* = 0.5, *M* = 0.5, K* = 1, *Gr* = 1, *Gm* = 10, *a* = 1, $t_{1}$ = 0.5.

**Figure 16:**
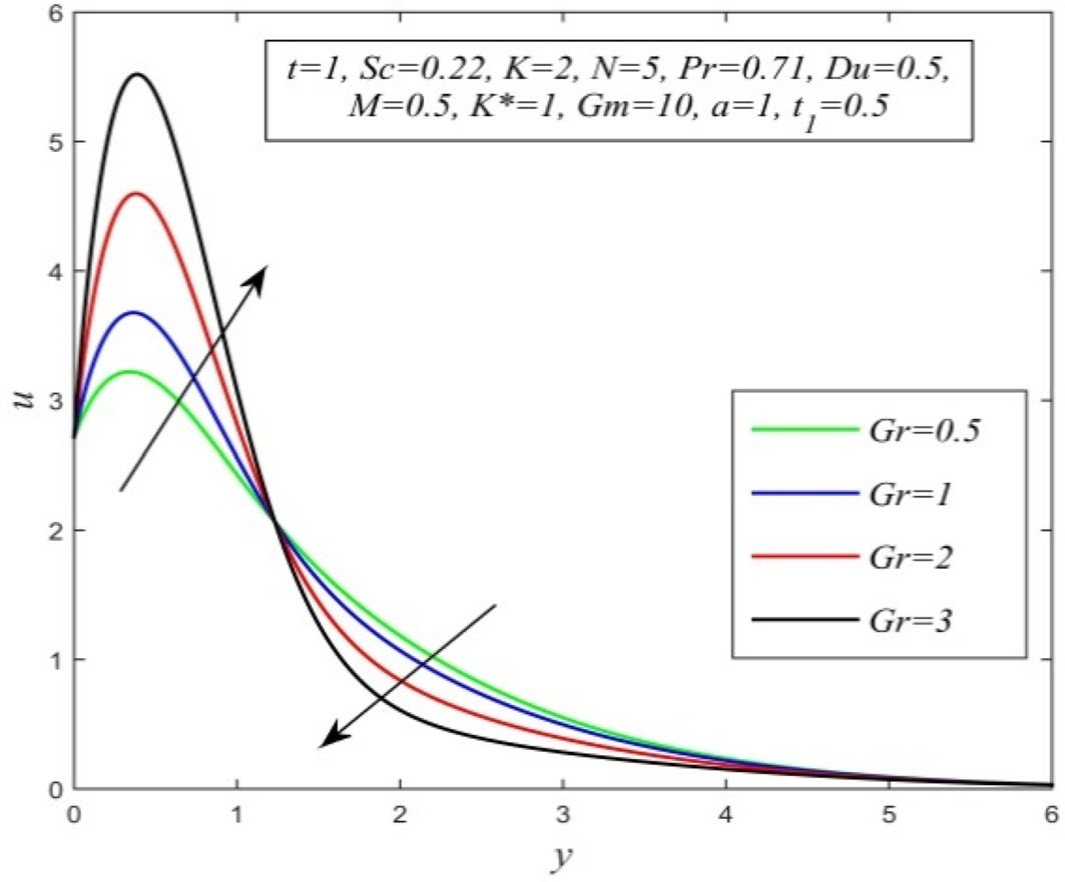
*u* versus *y* for different *Gr* and *t* = 1, *Sc* = 0.22, *K* = 2, *N* = 5, *Pr* = 0.71, *Du* = 0.5, *M* = 0.5, K* = 1, *Gm* = 10, *a* = 1, $t_{1}$ = 0.5.

**Figure 17:**
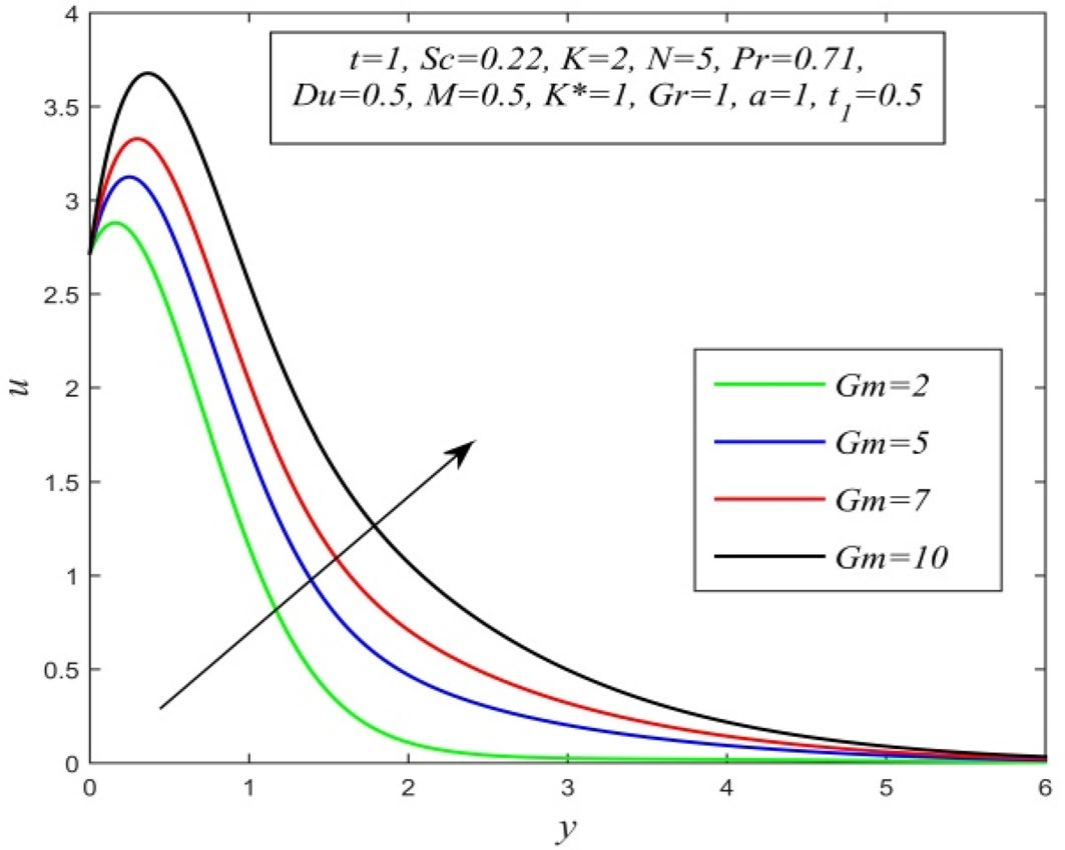
*u* versus *y* for different *Gm* and *t* = 1, *Sc* = 0.22, *K* = 2, *N* = 5, *Pr* = 0.71, *Du* = 0.5, *M* = 0.5, K* = 1, *Gr* = 1, *a* = 1, $t_{1}$ = 0.5.

**Figure 18:**
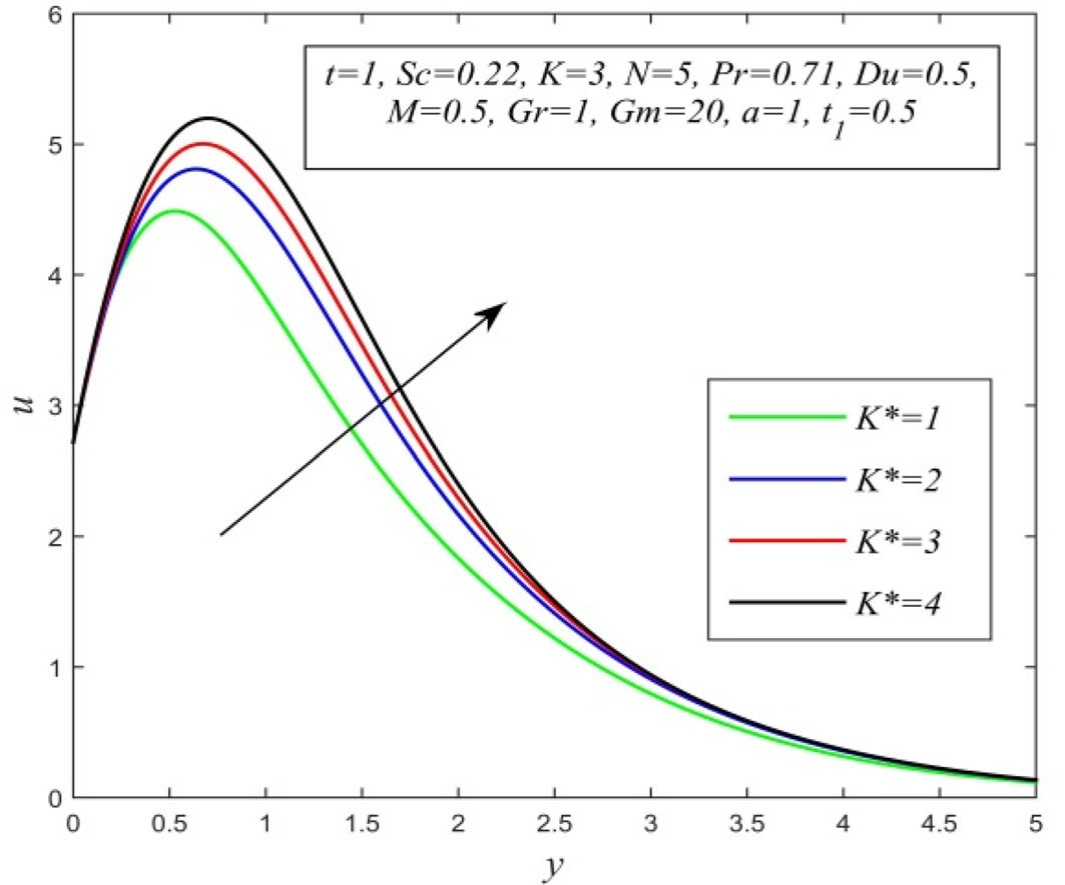
*u* versus *y* for different K* and *t* = 1, *Sc* = 0.22, *K* = 3, *N* = 5, *Pr* = 0.71, *Du* = 0.5, *M* = 0.5, *Gr* = 1, *Gm* = 20, *a* = 1, $t_{1}$ = 0.5.

**Figure 19:**
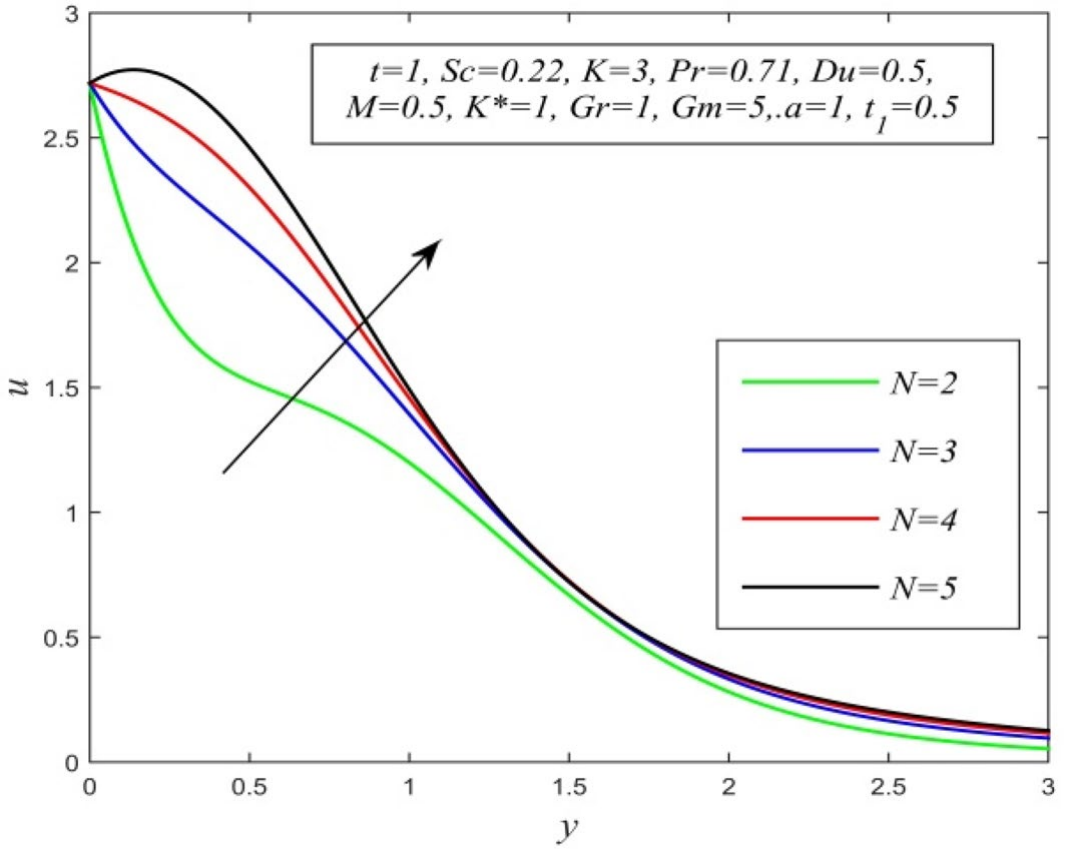
*u* versus *y* for different *N* and *t* = 1, *Sc* = 0.22, *K* = 3, *Pr* = 0.71, *Du* = 0.5, *M* = 0.5, K* = 1, *Gr* = 1, *Gm* = 5, *a* = 1, $t_{1}$ = 0.5.

**Figure 20:**
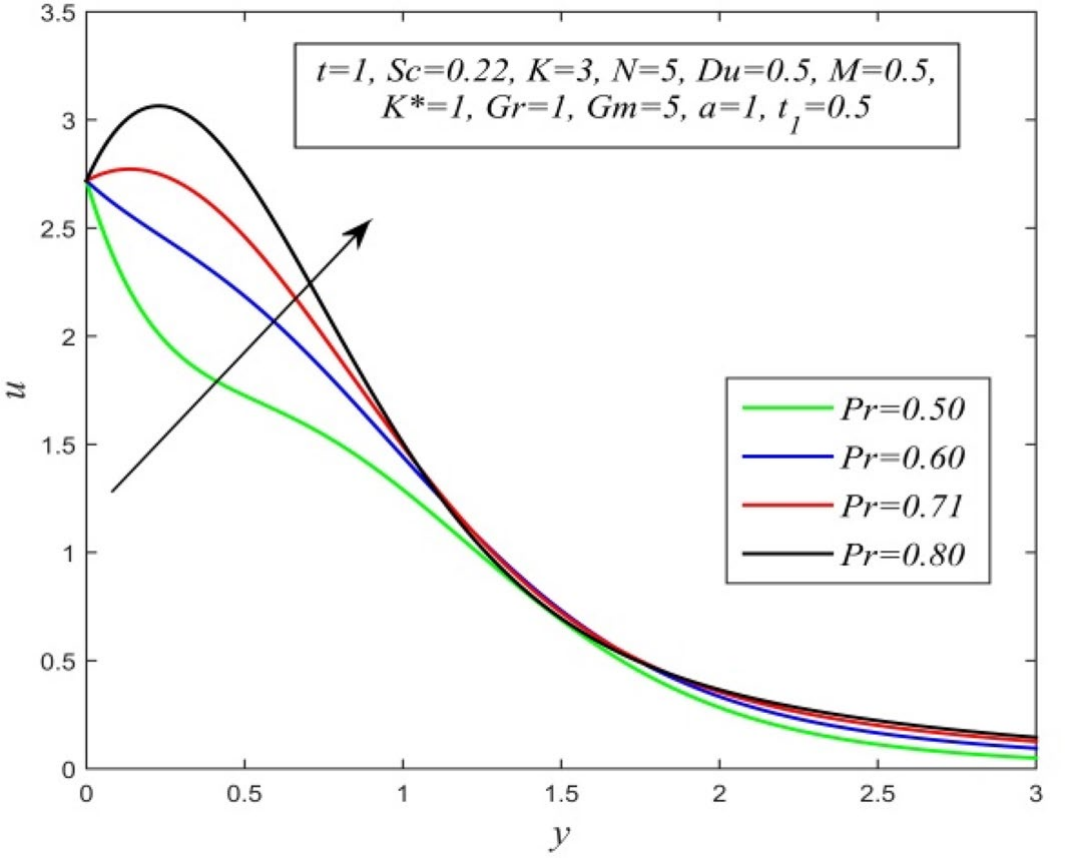
*u* versus *y* for different *Pr* and *t* = 1, *Sc* = 0.22, *K* = 3, *N* = 5, *Du* = 0.5, *M* = 0.5, K* = 1, *Gr* = 1, *Gm* = 5, *a* = 1, $t_{1}$ = 0.5.

**Figure 21:**
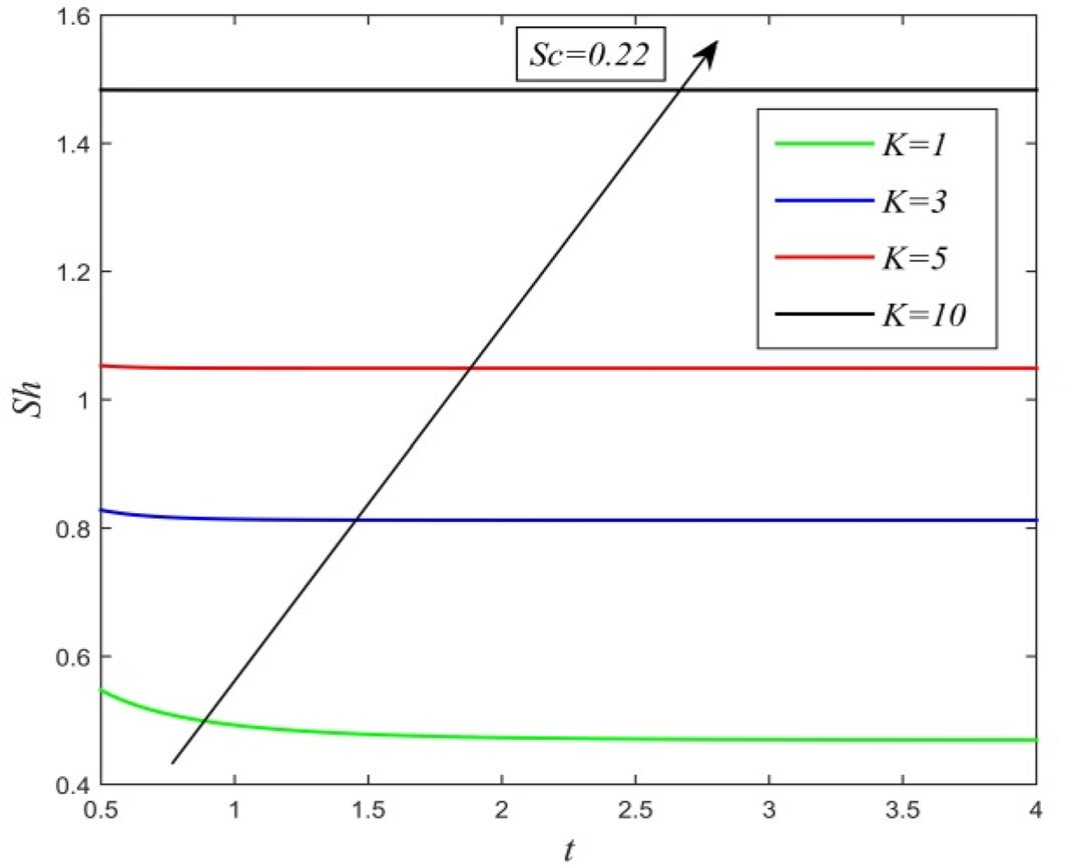
*Sh* versus *t* for different *K* and *Sc* = 0.22.

**Figure 22:**
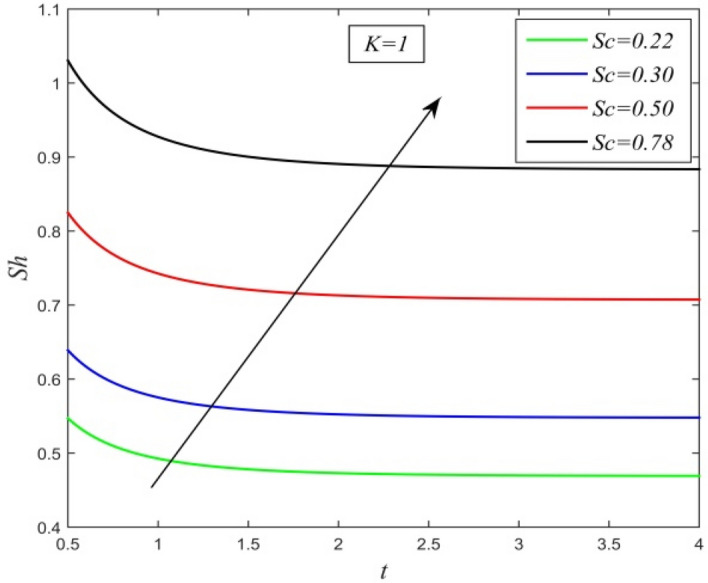
*Sh* versus *t* for different *Sc* and *K* = 1.

**Figure 23:**
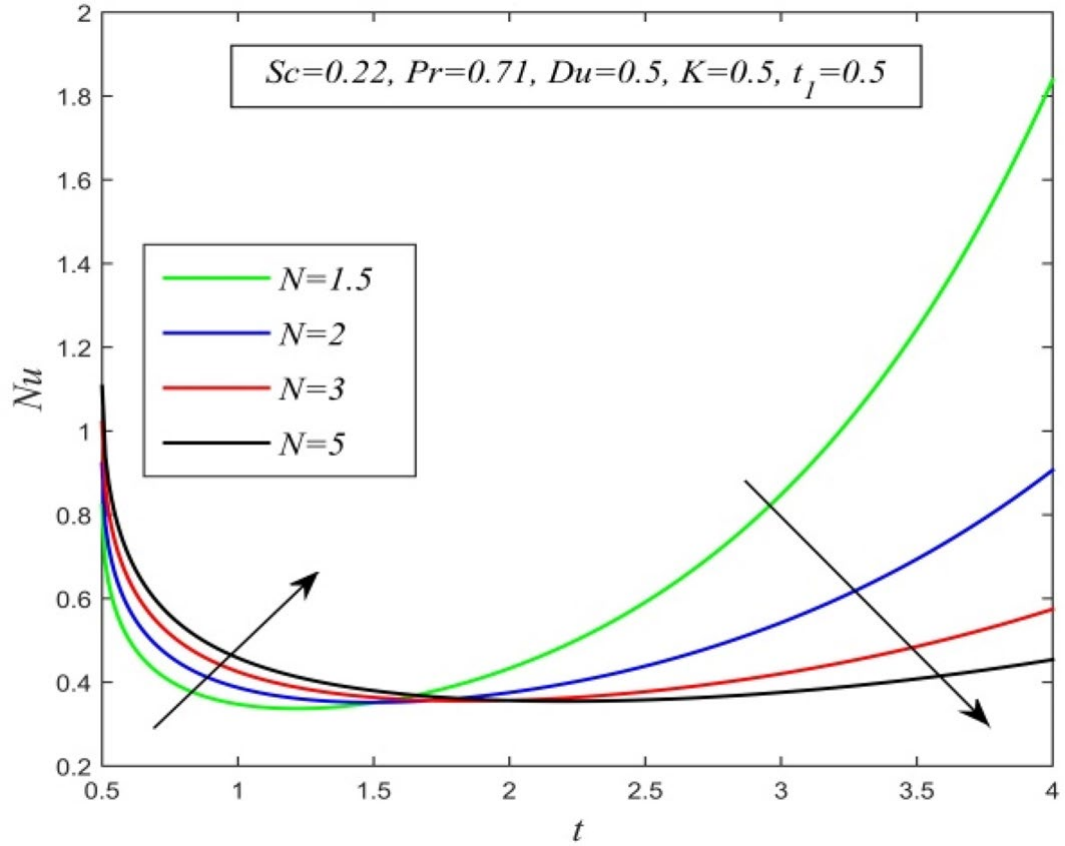
*Nu* versus *t* for different *N* and *Sc* = 0.22, *Pr* = 0.71, *Du* = 0.5, *K* = 0.5, $t_{1}$ = 0.5.

**Figure 24:**
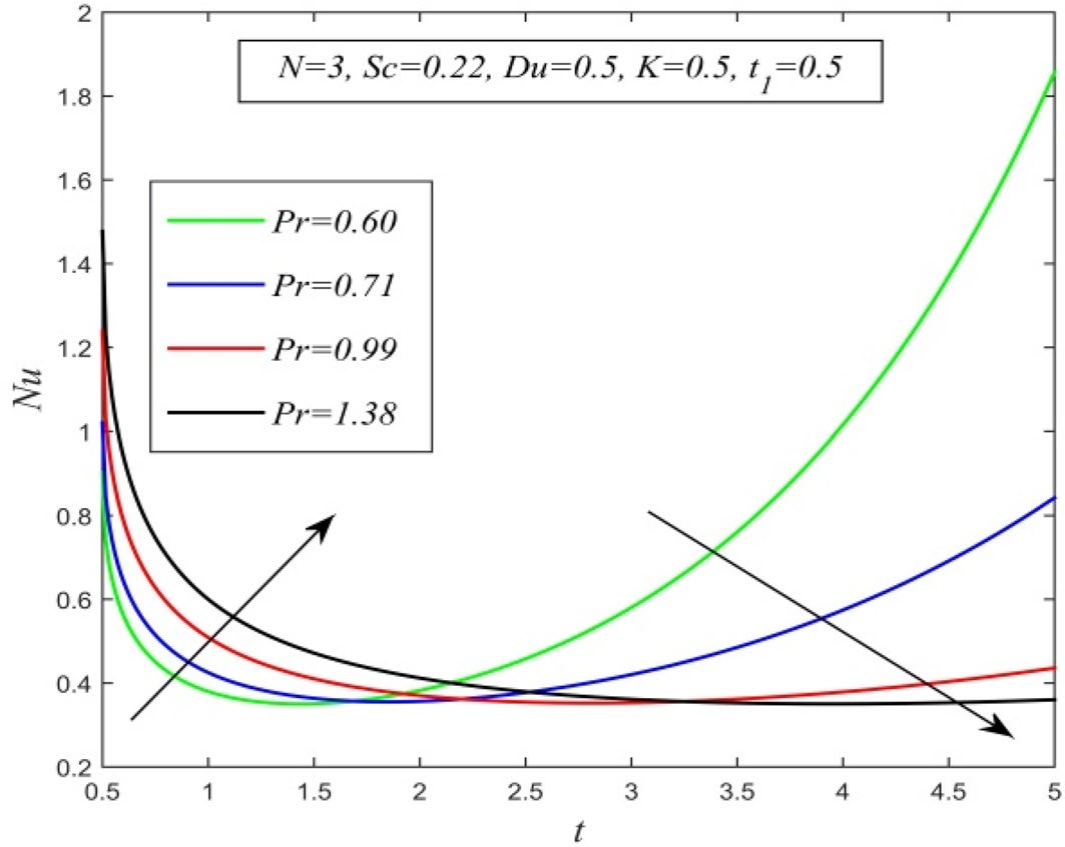
*Nu* versus *t* for different *Pr* and *Sc* = 0.22, *N* = 3, *Du* = 0.5, *K* = 0.5, $t_{1}$ = 0.5.

**Figure 25:**
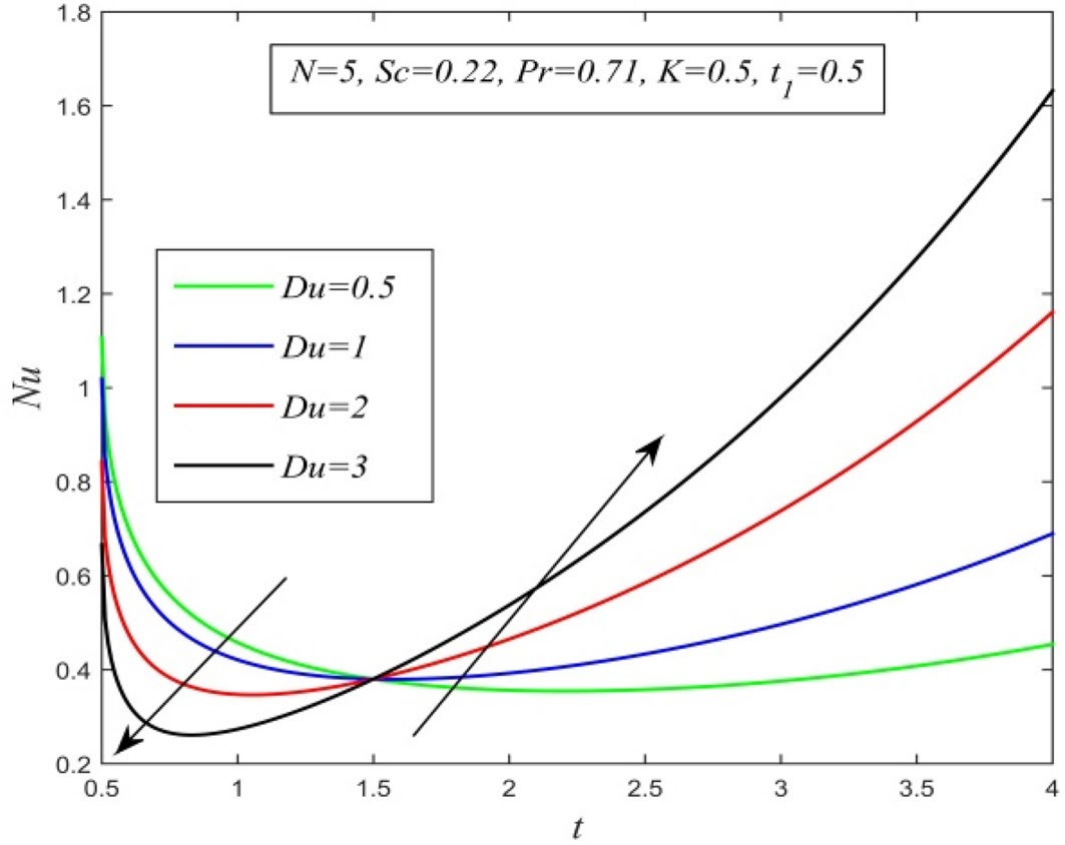
*Nu* versus *t* for different *Du* and *Sc* = 0.22, *N* = 5, *Pr* = 0.71, *K* = 0.5, $t_{1}$ = 0.5.

**Figure 26:**
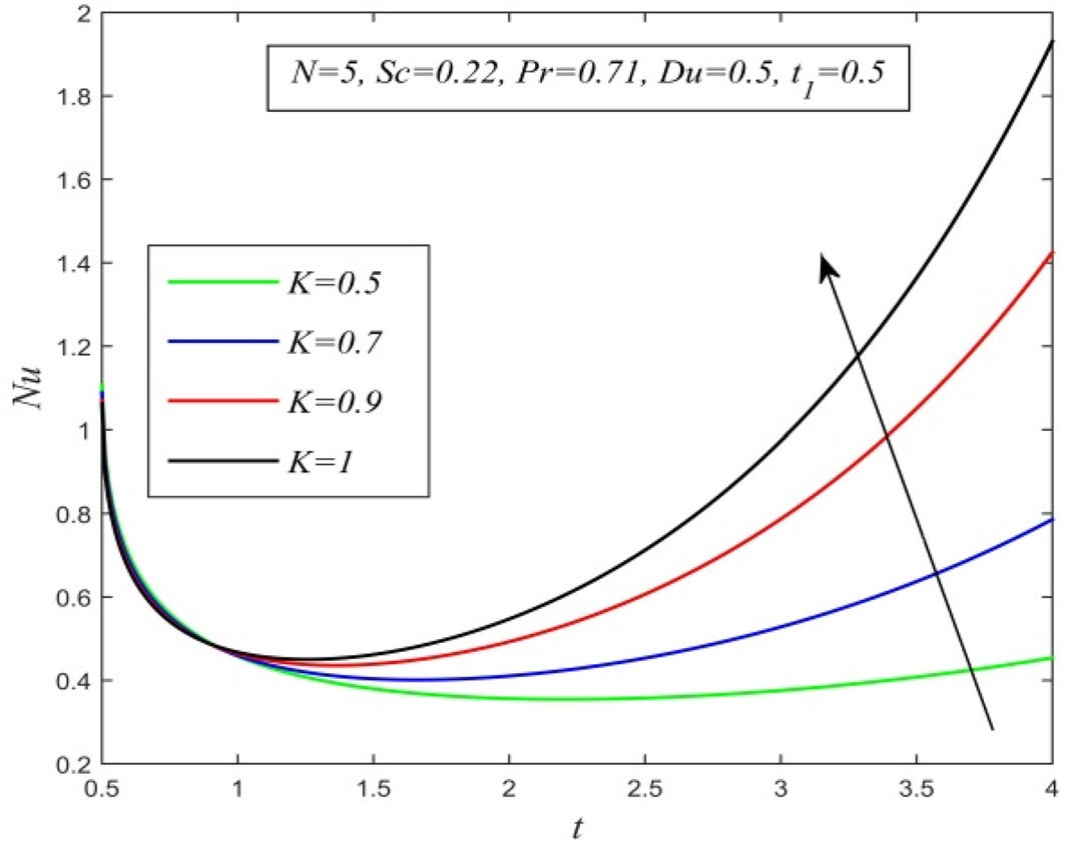
*Nu* versus *t* for different *K* and *Sc* = 0.22, *N* = 5, *Pr* = 0.71, *Du* = 0.5, $t_{1}$ = 0.5.

**Figure 27:**
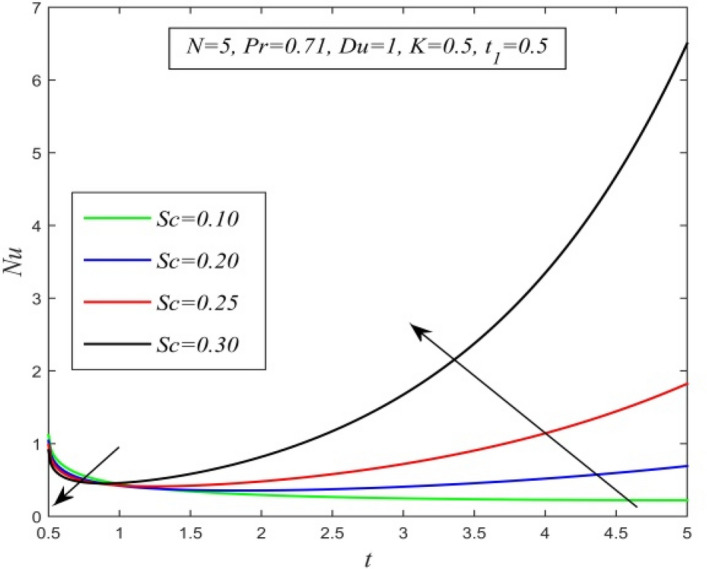
*Nu* versus *t* for different *Sc* and *N* = 5, *Pr* = 0.71, *Du* = 0.5, *K* = 0.5, $t_{1}$ = 0.5.

**Figure 28:**
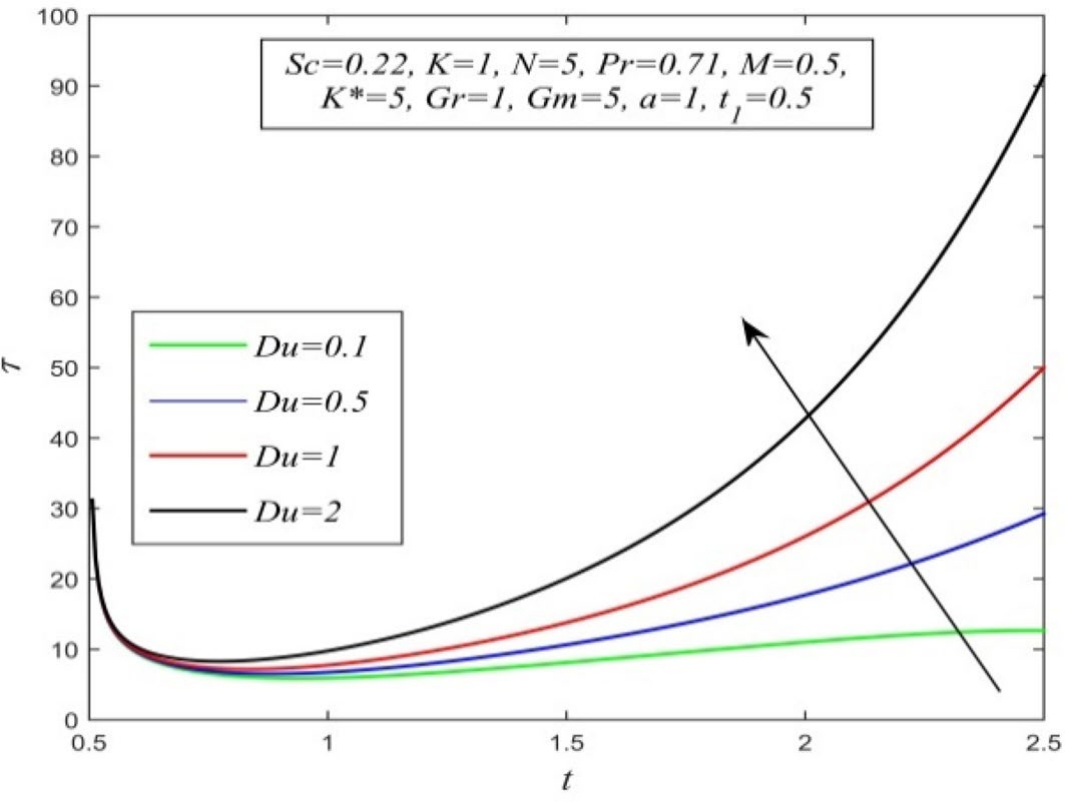
$\tau$ versus *t* for different *Du* and *Sc* = 0.22, *K* = 1, *N* = 5, *Pr* = 0.71, *M* = 0.5, K* = 5, *Gr* = 1, *Gm* = 5, *a* = 1, $t_{1}$ = 0.5.

**Figure 29:**
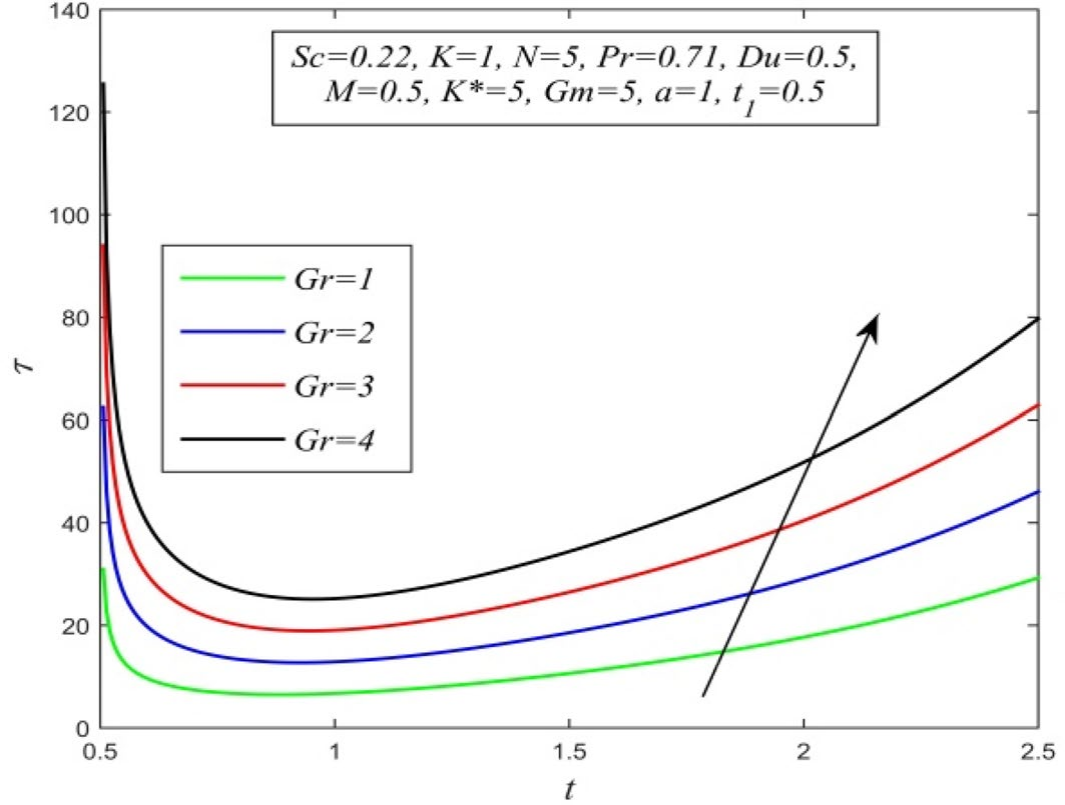
$\tau$ versus *t* for different *Gr* and *Sc* = 0.22, *K* = 1, *N* = 5, *Pr* = 0.71, *Du* = 0.5, *M* = 0.5, K* = 5, *Gm* = 5, *a* = 1, $t_{1}$ = 0.5.

**Figure 30:**
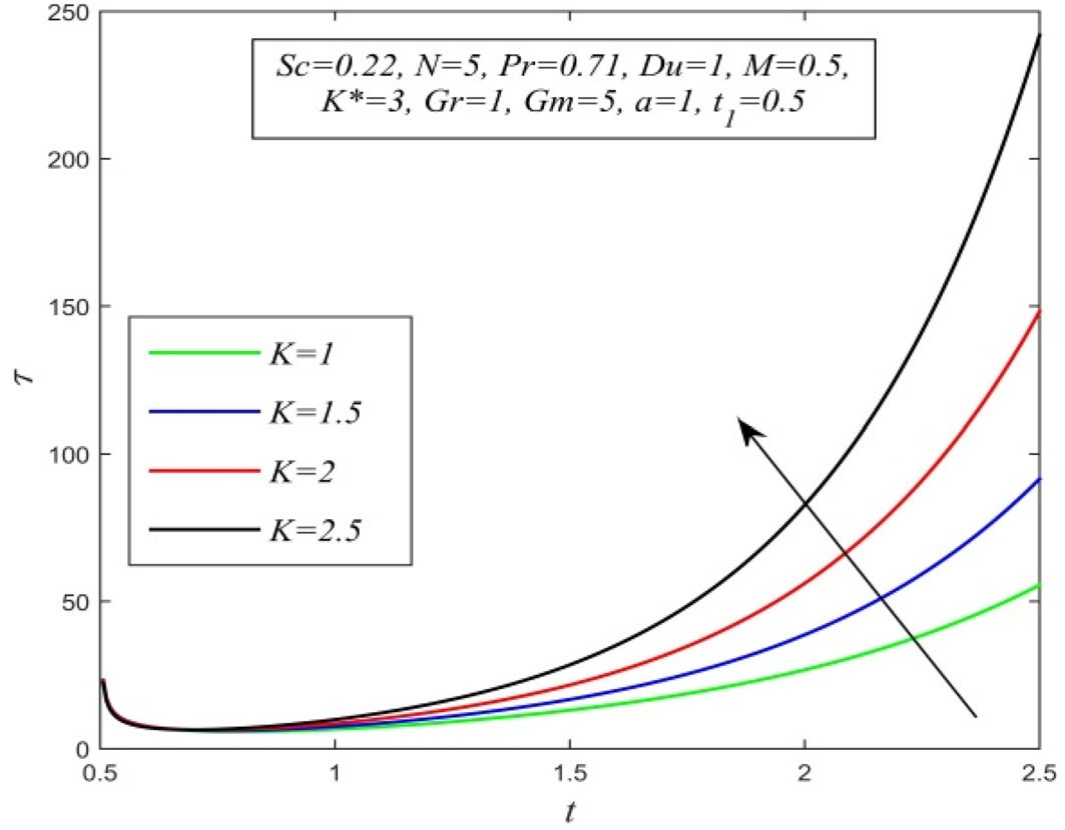
$\tau$ versus *t* for different *K* and *Sc* = 0.22, *N* = 5, *Pr* = 0.71, *Du* = 1, *M* = 0.5, K* = 3, *Gr* = 1, *Gm* = 5, *a* = 1, $t_{1}$ = 0.5.

**Figure 31:**
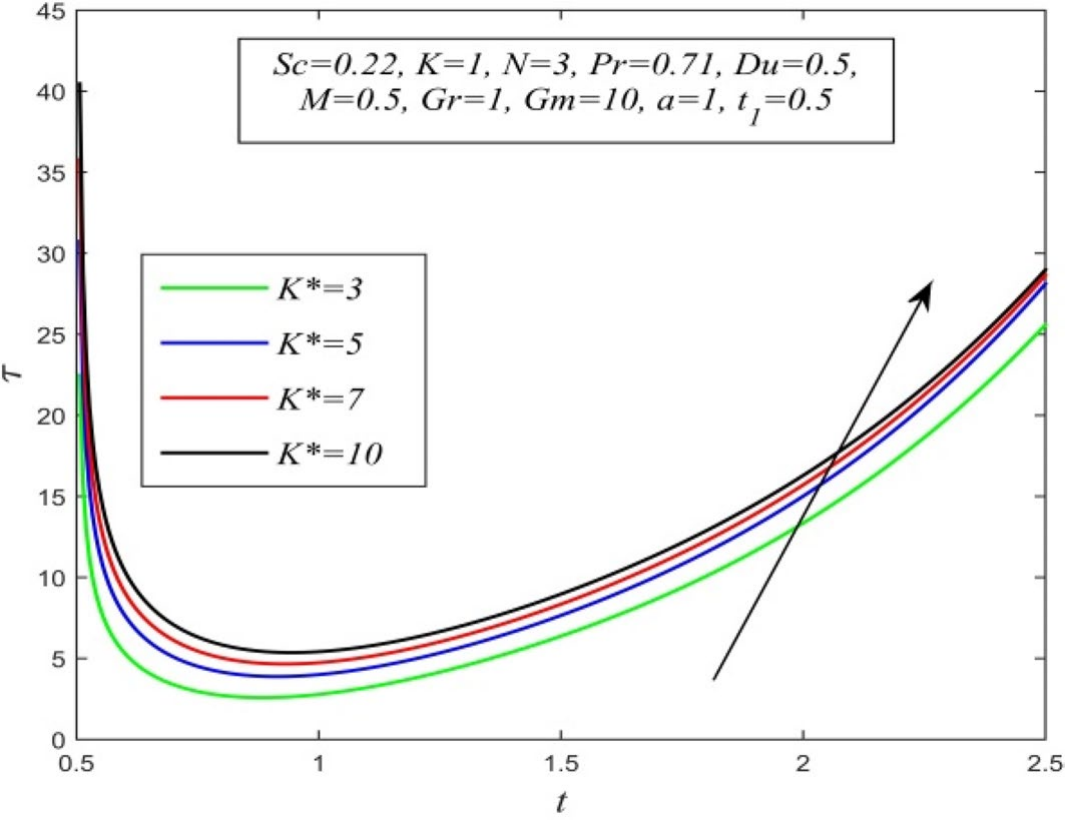
$\tau$ versus *t* for different K* and *Sc* = 0.22, *K* = 1, *N* = 3, *Pr* = 0.71, *Du* = 0.5, *M* = 0.5, *Gr* = 1, *Gm* = 10, *a* = 1, $t_{1}$ = 0.5.

**Figure 32:**
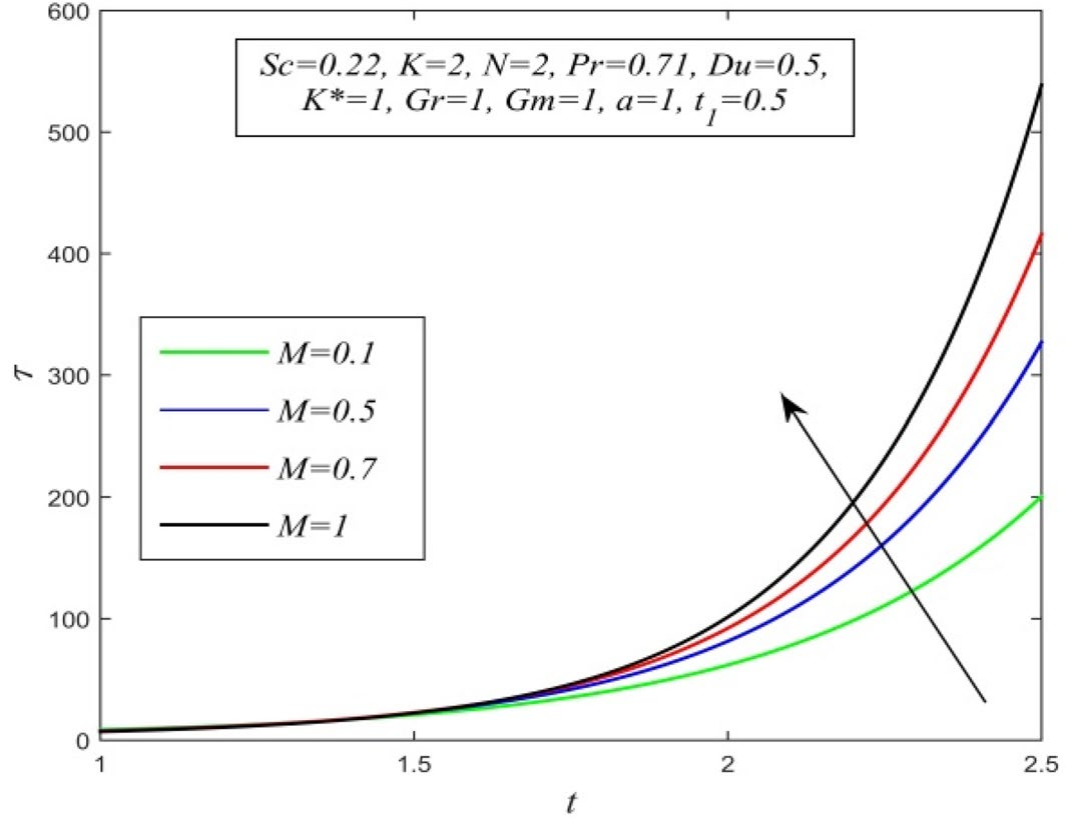
$\tau$ versus *t* for different *M* and *Sc* = 0.22, *K* = 2, *N* = 2, *Pr* = 0.71, *Du* = 0.5, K* = 1, *Gr* = 1, *Gm* = 1, *a* = 1, $t_{1}$ = 0.5.

**Figure 33:**
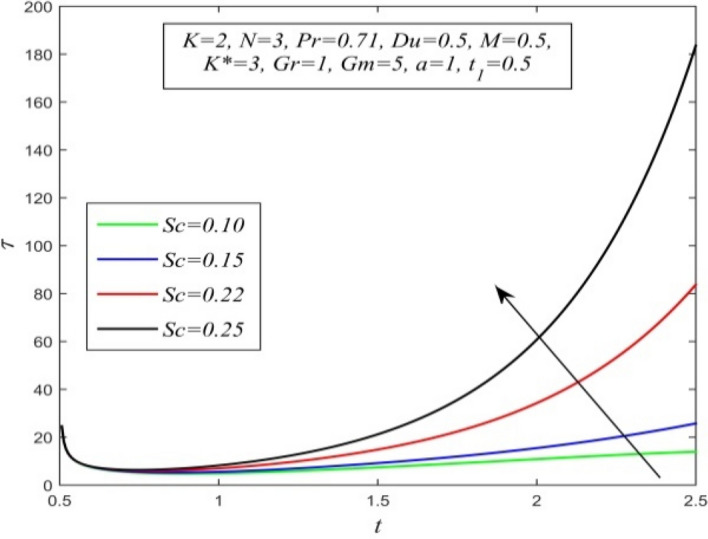
$\tau$ versus *t* for different *Sc* and *K* = 2, *N* = 3, *Pr* = 0.71, *M* = 0.5, *Du* = 0.5, K* = 3, *Gr* = 1, *Gm* = 5, *a* = 1, $t_{1}$ = 0.5.

**Figure 34:**
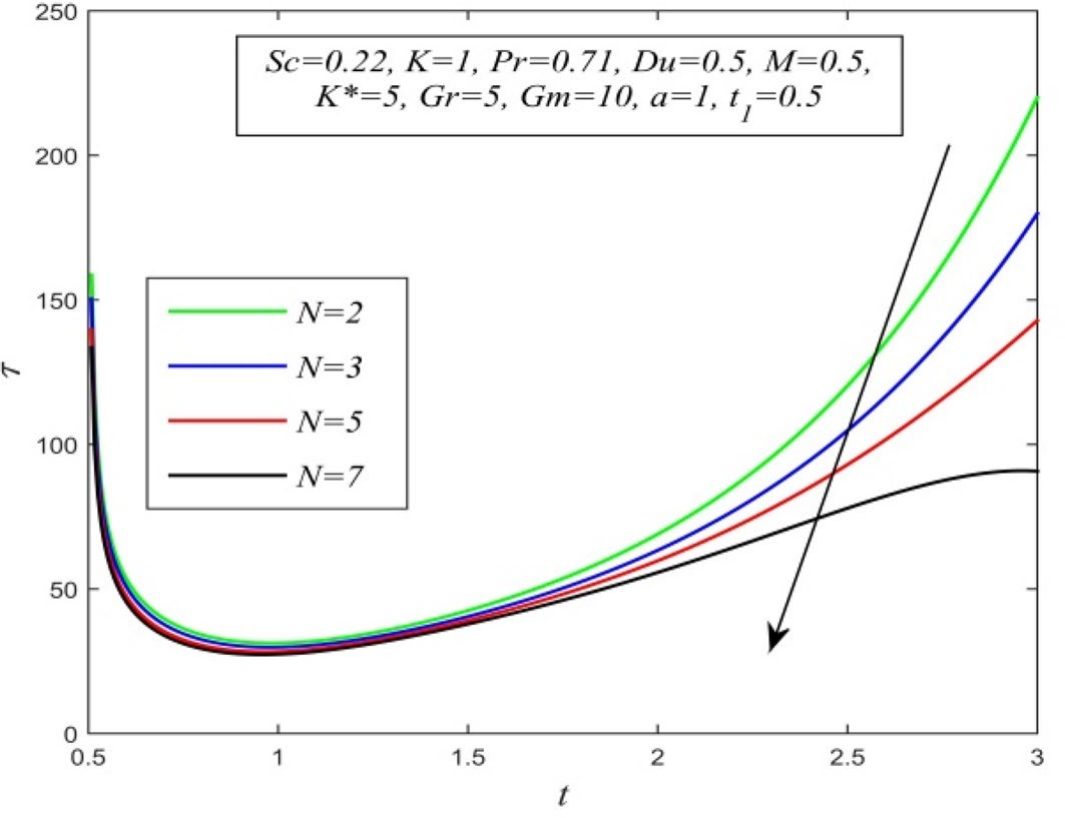
$\tau$ versus *t* for different *N* and *Sc* = 0.22, *K* = 1, *Pr* = 0.71, *M* = 0.5, *Du* = 0.5, K* = 5, *Gr* = 5, *Gm* = 10, *a* = 1, $t_{1}$ = 0.5.

**Figure 35:**
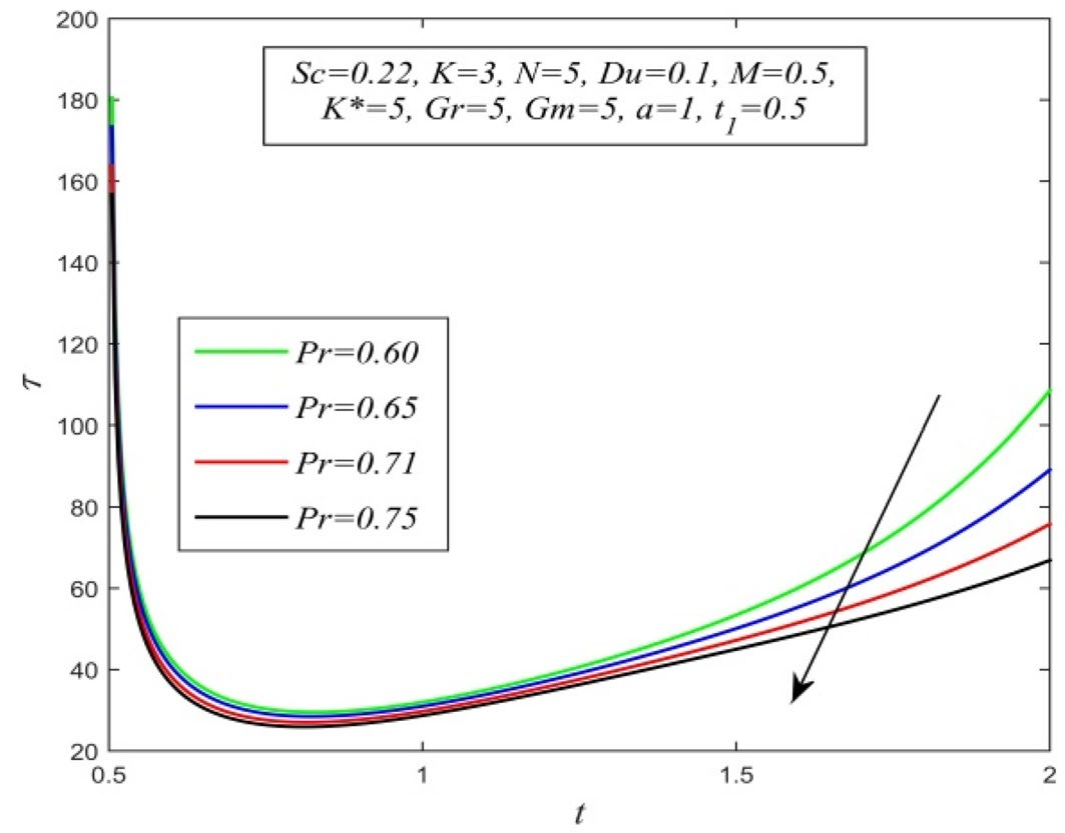
$\tau$ versus *t* for different *Pr* and *Sc* = 0.22, *K* = 3, *N* = 5, *M* = 0.5, *Du* = 0.1, K* = 5, *Gr* = 5, *Gm* = 5, *a* = 1, $t_{1}$ = 0.5.

Figures 2, 3 and 4 display the variation of concentration field versus normal co-ordinate y. Figure 2 admits that the concentration field keeps on increasing with time. Figure 3 reveals that there is a comprehensive fall in the concentration field for increasing chemical reaction parameter. A faster chemical reaction consumes chemical substances present in the fluid rapidly and as a result concentration of the fluid declines. The behaviour of concentration profiles for various fluids such as hydrogen (Sc = 0.22), helium (Sc = 0.30), water vapour (Sc = 0.60) and ammonia (Sc = 0.78) are demonstrated in Fig. 4. It suggests that a higher Schmidt number lowers the concentration field. Thus higher mass diffusivity hikes the concentration field.

Figures 5, 6, 7, 8, 9 and 10 illustrate the variation of temperature field versus normal co-ordinate y. Figure 5 suggests that the temperature field escalates with time. Figure 6 shows that the temperature field upsurges with increment in chemical reaction parameter. Increasing chemical reaction parameter upsurges collision between fluid molecules and as a result temperature of fluid hikes. Figure 7 displays that increasing the Dufour number hikes temperature field. An increment in the Dufour number indicates a comprehensive rise in concentration gradient over temperature gradient. Hence, increasing concentration gradient upsurges the temperature field. Figure 8 suggests that the temperature field elevates with an uplift in Schmidt number. Thus, the temperature field decreases with increasing mass diffusivity. The temperature field decelerates with increasing radiation parameter as noticed in Fig. 9. It is in agreement with the fact that radiation tends to decline temperature. The nature of temperature profiles for various fluids such as oxygen (Pr = 0.60), air (Pr = 0.71), ammonia (Pr = 1.38) etc. are demonstrated in Fig. 10. It shows that the temperature field falls with ascending values of the Prandtl number. This informs that the temperature field accelerates with higher thermal diffusivity.

Figures 11, 12, 13, 14, 15, 16, 17, 18, 19 and 20 depict the variation of velocity field versus normal co-ordinate y. Figure 11 reveals that as time progresses, the velocity field increases. Figure 12 admits that the velocity field declines considerably as the Dufour number rises. Consequently, a large concentration gradient relative to the temperature gradient results in a dip in the velocity field. Figure 13 shows that velocity reduces with increasing chemical reaction parameter. This is because increasing chemical reaction parameter accelerates the process of collision between fluid molecules and as a result, kinetic energy is lost. Velocity falls with increasing magnetic parameter as noticed in Fig. 14. Application of transverse magnetic field produces a resistive force known as Lorentz force, which slows down fluid velocity. Figure 15 exhibits that increasing Schmidt number decrease velocity field. Thus, high mass diffusivity escalates fluid velocity. Velocity field upsurges in a thin layer adjacent to the plate and its nature take reverse turn outside the layer as thermal Grashof number upsurges as demonstrated in Fig. 16. So, thermal buoyancy force hikes velocity in a small layer surrounding the plate but lowers velocity outside the layer. Velocity rises with increment in solutal Grashof number as noticed in Fig. 17. Thus, solutal buoyancy force upsurges velocity. Hence higher mass diffusivity raises velocity field but increasing thermal diffusivity reduces velocity. Increasing porosity parameter means the fluid gets more free space to flow. As a result fluid velocity hikes. This phenomenon is reflected in Fig. 18. Increasing radiation parameter accelerates fluid velocity as observed in Fig. 19. The reason behind it is that when the radiation increases, chemical bonding between the fluid molecules becomes weak so that velocity hikes. Figure 12 shows that ascending values of Prandtl number uplift velocity. Thus, higher thermal diffusivity diminishes velocity.

Figures 21 and 22 demonstrate the variation of Sherwood number versus time *t*. Sherwood number increases with increment in chemical reaction parameter as noticed in Fig. 11. From Fig. 22, it is observed that increasing Schmidt number upsurges Sherwood number. This result establishes the fact that higher mass diffusivity accelerates the process of mass transfer from the plate to the fluid.

Figures 23, 24, 25, 26 and 27 exhibit the variation of Nusselt number versus time t. Nusselt number increases for a small time but decreases thereafter for increasing radiation parameter as noticed in Fig. 24. Thus, radiation increases the rate of heat transfer from the plate to the fluid for a small time and decreases afterward. Figure 26 shows that the Nusselt number hikes for a small time but declines thereafter with ascending values of the Prandtl number. So, higher thermal diffusivity lessens the rate of heat transfer for a small time but increases as time progresses. From Fig. 25 and Fig. 27, it is observed that the Nusselt number declines for a small time but upsurges thereafter with increment in Dufour number and Schmidt number respectively. Figure 26 shows that higher chemical reaction parameter hikes Nusselt number. Increasing chemical reaction parameter suggests a hike in heat generation. So, the process of heat transfer is accelerated.

Variations of skin friction versus time t are demonstrated in Figs. 28, 29, 30, 31, 32, 33, 34 and 35. Figure 28 admits that there is a comprehensive rise in skin friction as Dufour number hikes. Thus, the concentration gradient generates more frictional resistance compared to the temperature gradient. Skin friction uplifts with increment in thermal Grashof number as noticed in Fig. 29. Thus, thermal buoyancy force hikes frictional resistivity at the plate. Skin friction hikes with an upsurge in both chemical reaction parameter and porosity parameter as shown in Fig. 30 and Fig. 31 respectively.Fig. 32 reveals that increasing magnetic parameter raises skin friction. Hence Lorentz force accelerates frictional resistivity of the plate. Higher Schmidt number hikes skin friction as displayed in Fig. 33. Therefore, increasing mass diffusivity lowers the frictional resistance of the plate.Fig. 34 and Fig. 35 give us an idea that enhancement in radiation parameter and Prandtl number lowers skin friction.

**Figure 36:**
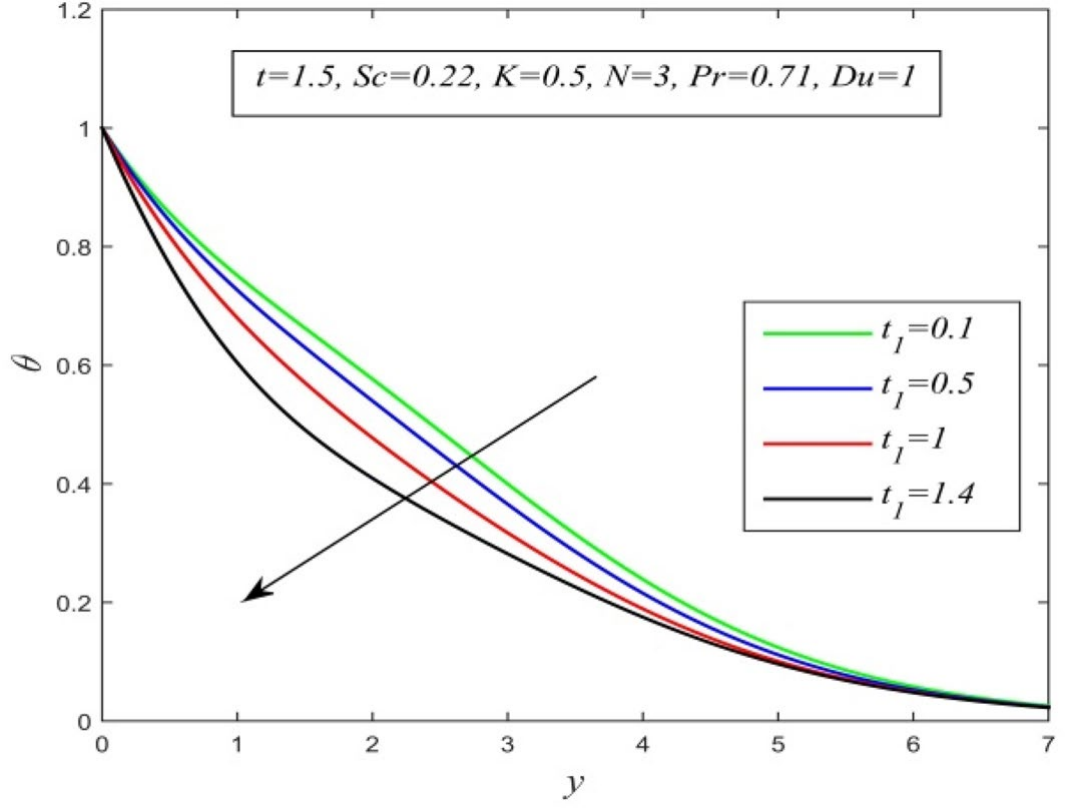
$\theta$ versus *y* for different $t_{1}$ and *t* = 1.5, *Sc* = 0.22, *K* = 0.5, *N* = 3, *Pr* = 0.71, *Du* = 1.

**Figure 37:**
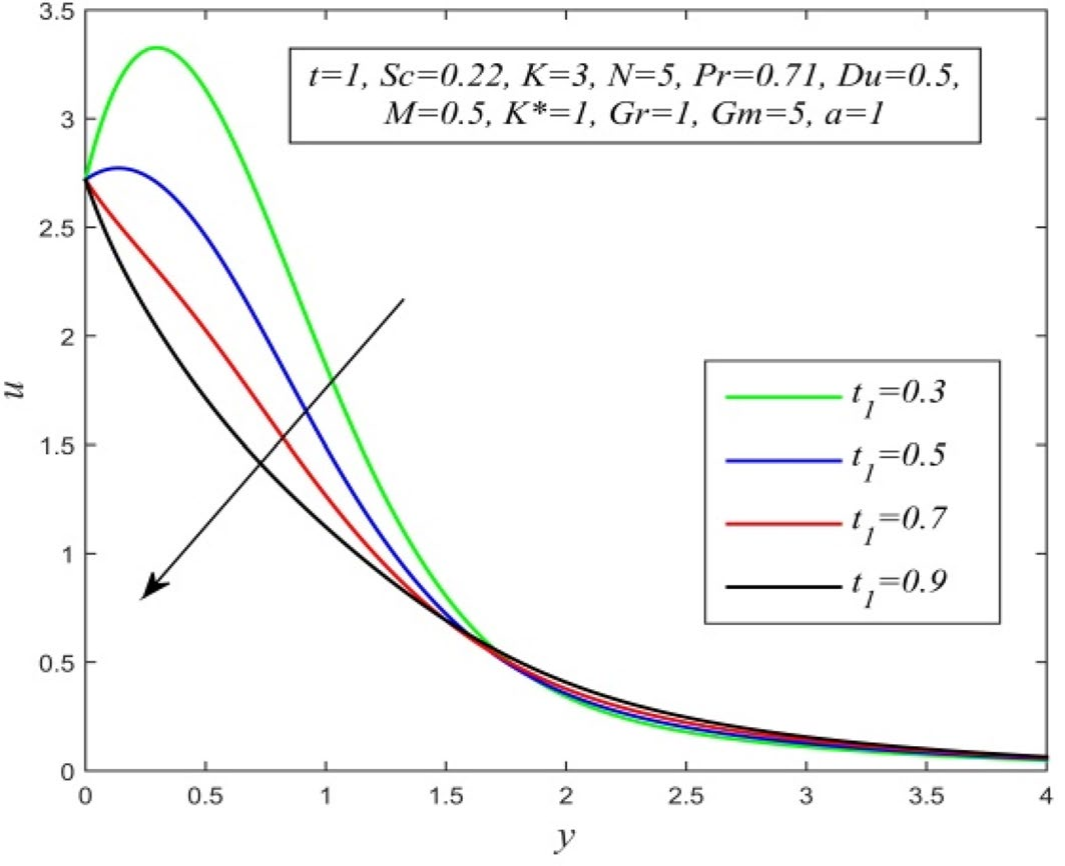
*u* versus *y* for different $t_{1}$ and *t* = 1, *Sc* = 0.22, *K* = 3, *N* = 5, *Pr* = 0.71, *Du* = 0.5, *M* = 0.5, K* = 1, *Gr* = 1, *Gm* = 5, *a* = 1.

**Figure 38:**
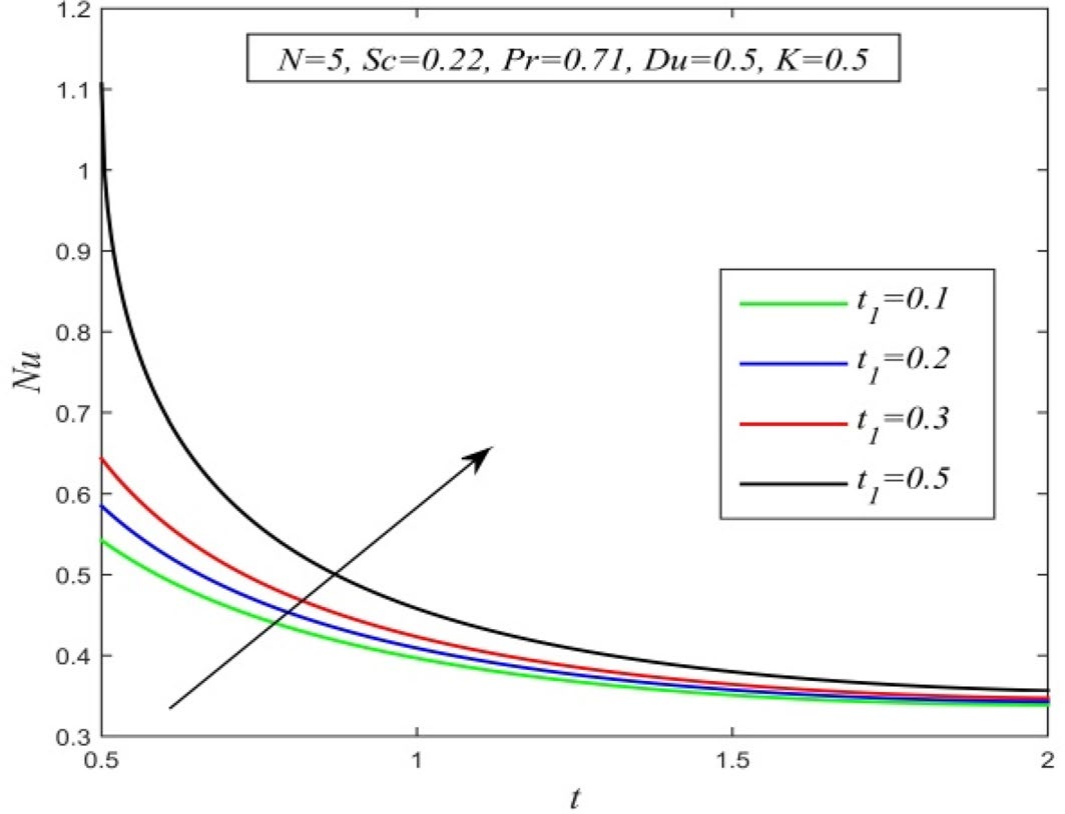
*Nu* versus *t* for different $t_{1}$ and *N* = 5, *Sc* = 0.22, *K* = 0.5, *Pr* = 0.71, *Du* = 0.5.

**Figure 39:**
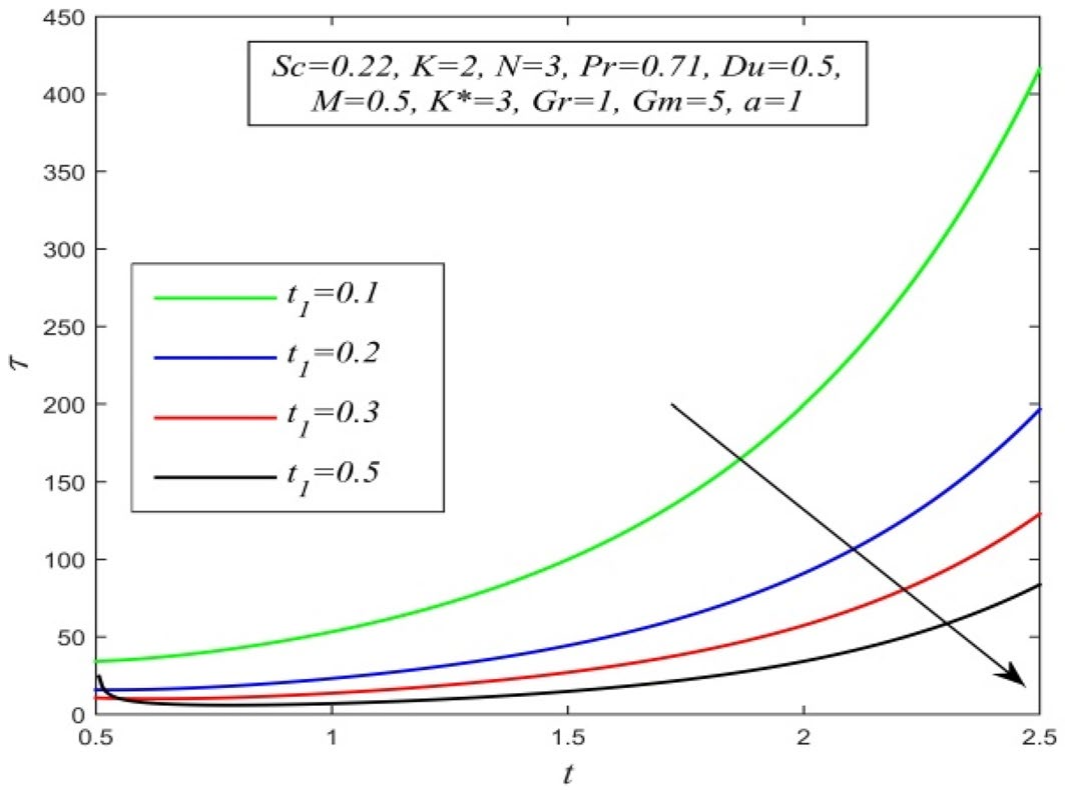
$\tau$ versus *t* for different $t_{1}$ and *Sc* = 0.22, *K* = 2, *N* = 3, *Pr* = 0.71, *Du* = 0.5, *M* = 0.5, K* = 3, *Gr* = 1, *Gm* = 5, *a* = 1.

Figure 36 and Fig. 37 reveal that ascending critical time for rampedness lowers both temperature and velocity of the fluid respectively. Thus, arbitrary ramped temperature has inverse effect on both temperature and velocity fields. It is observed from Fig. 38 that increasing critical time for rampedness hikes Nusselt number. This means that arbitrary ramped temperature effect has a tendency to accelerate the rate of heat transfer from the plate to the fluid. Figure 39 shows that increasing critical time for rampedness declines skin friction. Thus arbitrary ramped temperature weakens the rate of momentum transfer from the plate to the fluid.

Numerical values of Nusselt number *Nu* against different time *t*, Dufour number *Du* and radiation parameter are analyzed in Table1. It is observed that for a small time, the Nusselt number decreases with increment in Dufour number but its behavior reverses as time progresses. An opposite behavior is noticed for increasing radiation parameter. This asserts that a high concentration gradient decelerates but radiation accelerates the process of heat transfer from the plate to the fluid. This is in complete agreement with our results from Fig. 23 and Fig. 25.Numerical values of skin friction $\tau$ against different time *t*, chemical reaction parameter *K*, radiation parameter *N*, Dufour number *Du*, thermal Grashof number *Gr* and solutal Grashof number *Gm* are demonstrated in Table2. It is noticed that ascending values of time, chemical reaction parameter, Dufour number, and thermal Grashof number hike skin friction whereas ascending values of radiation parameter and solutal Grashof number declines the value of skin friction. This is in accordance with our result from Fig. 30 and Fig. 28, Fig. 29 and Fig. 34 respectively.

**Table 1:** Computational values of Nusselt number for various *t,* *Du* and *N* when *Pr* = 0.71, *Sc* = 0.22, K = 0.5, $t_{1}$ = 0.5.

*t*	*Du*	*N*	*Nu*
0.5	0.5	5	1.1016
0.5	1	5	1.0184
0.5	1.5	5	0.9302
2	0.5	5	0.3564
2	1	5	0.3926
2	1.5	5	0.4288
0.5	0.5	2	0.9218
0.5	0.5	3	1.0204
0.5	0.5	4	1.0730
2	0.5	2	0.3753
2	0.5	3	0.3565
2	0.5	4	0.3552

**Table 2:** Computational values of skin friction for various *t,* *K,* *N,* *Du,* *Gr* and *Gm* when *Pr* = 0.71, *Sc* = 0.22, *a* = 1*,* *M = *0.5, K* = 0.5, $t_{1}$ = 0.5.

*t*	*K*	*N*	*Du*	*Gr*	*Gm*	$\tau$
1	1	5	0.5	1	1	9.1499
1.5						13.2425
2						20.4193
1	2	5	0.5	1	1	9.9629
	3					11.2420
	5					16.8475
1	1	2	0.5	1	1	9.7355
		5				9.1499
		7				8.9585
1	1	5	1	1	1	10.1642
			2			14.2214
			3			18.2786
1	1	5	0.5	1	1	9.1499
				3		21.4736
				5		33.7974
1	1	5	0.5	1	1	9.1499
					3	7.9392
					5	6.7286

## Comparison of result

To check the validity of our result, we have compared one of our results with Seth et al.^49^ who considered the unsteady free convective MHD flow of a chemically reactive, radiative flow past a moving vertical plate immersed in a porous medium. In absence of Dufour and chemical reaction effects and for vanishing Schmidt number (i.e., *Du* = *0,* *K* = *0* and *Sc* = *0*), expression of temperature field of the present problem is

$$\theta = \theta_{1,1}$$

**Figure 40:**
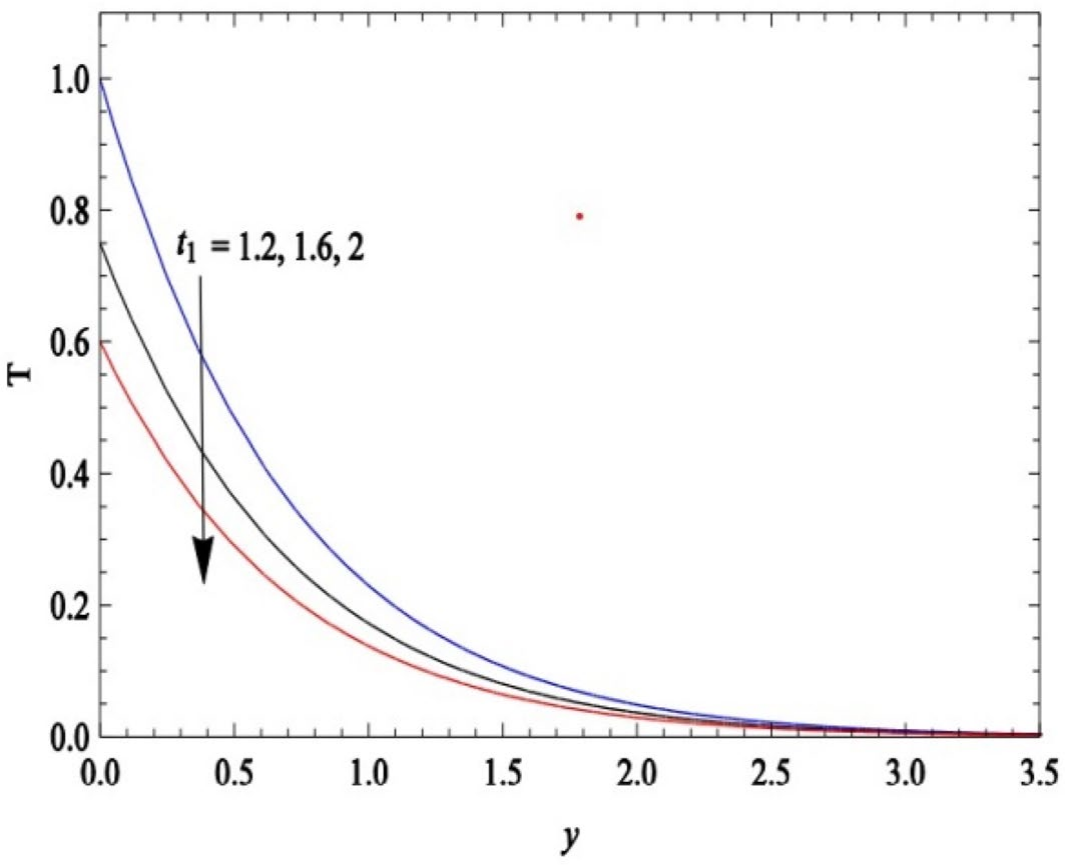
Scanned graph of temperature field versus y for different $t_{1}$ when *t* = *1.2,* *N* = *2,* *Pr* = *0.71* drawn by Seth et al.^49^.

**Figure 41:**
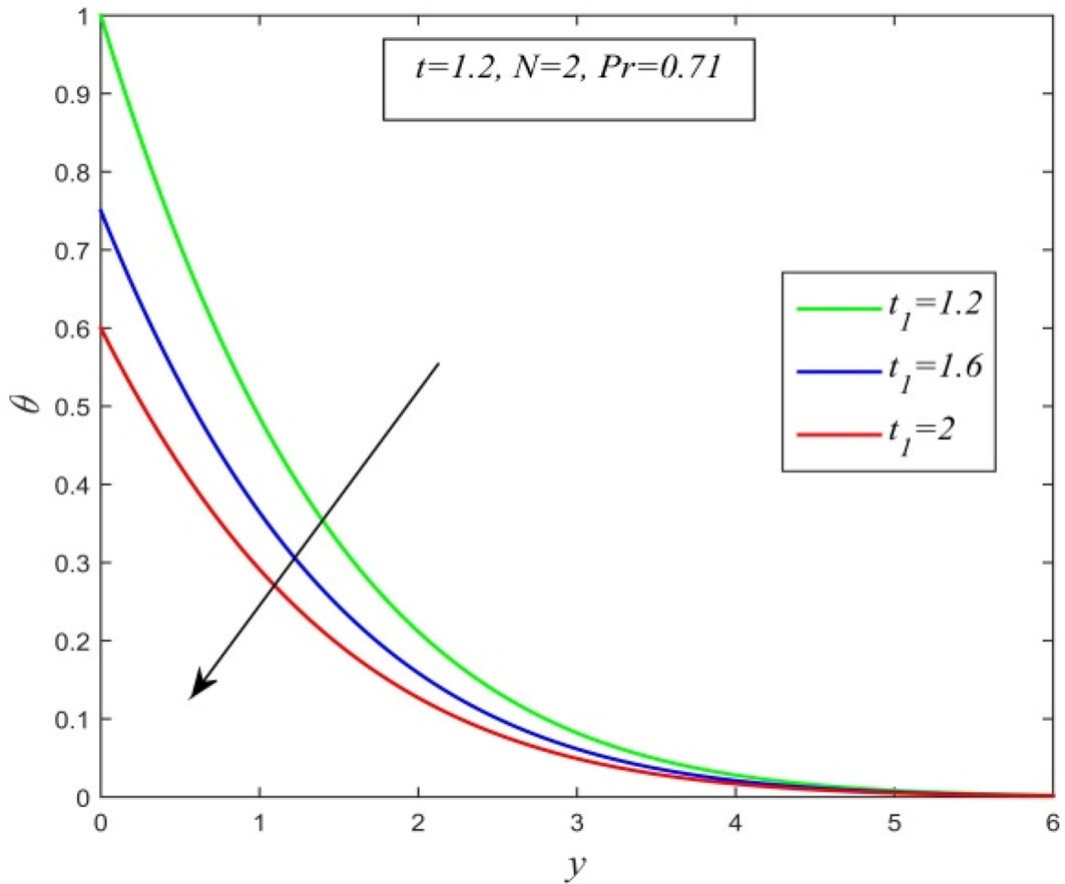
temperature field versus y for different $t_{1}$ when *t* = *1.2,* *N* = *2,* *Pr* = *0.71*, *Sc* = *0,* *Du* = *0,K* = *0.*

Figure 40 and Fig. 41 display the temperature field versus normal co- ordinate y for different $t_{1}$ obtained by Seth et al.^49^ and present authors respectively. Both figures uniquely expresses the fact that temperature field declines for ascending values of critical time of rampedness. Hence, an excellent agreement of results between present authors and Seth et al.^49^ is observed.

Table 3 display the variation of Sherwood number for different *K*, *Sc* and *t* obtained by Asogwa et al.^50^, Seth et al.^51^, Kataria and Patel^52^ and present authors respectively. This table indicates that current study is in line with the results obtained by these authors.

**Table 3:** Comparison of computational values of Sherwood number for various *K,* *Sc* and *t* obtained by Asogwa et.al^50^, Seth et. Al^51^, Kataria and Patel^52^ and present authors.

K	Sc	T	Asogwa et. al^50^ (isothermal condition)	Seth et. al^51^ (isothermal condition)	Kataria and Patel^52^ (isothermal condition)	Present study (isothermal condition)
5	0.66	0.4	1.8320	1.8320	1.8320	1.8320
5.1	0.66	0.4	1.8493	1.8493	1.8493	1.8493
5.2	0.66	0.4	1.8664	1.8664	1.8664	1.8664
5	0.7	0.4	1.8867	1.8867	1.8867	1.8867
5	0.8	0.4	2.0170	2.0170	2.0170	2.0170
5	0.66	0.5	1.8238	1.8238	1.8238	1.8238
5	0.66	0.6	1.8201	1.8201	1.8201	1.8201
5	1.24	0.4	2.5111	–	–	2.5117
5	2.01	0.4	3.1971	–	–	3.1971

## Conclusion

The prime purpose of the present work was to study exclusively the effects of radiation, chemical reaction and Diffusion thermo effect of an unsteady MHD flow past a moving vertical plate embedded in a porous medium with ramped temperature. The behavioral study of flow and transport characteristics under the action of different parameters was carried out with aid of graphs. The prominent outcomes of the present work are as follows:

i. Velocity field, concentration field, and temperature field accelerate with time.
ii. Fluid gets thinner rapidly as chemical reaction parameter and Schmidt number hikes.
iii. Radiation and Lorentz force resists fluid velocity.
iv. Higher mass diffusivity results in a fall in Nusselt Number, Sherwood number, and skin friction.
v. Radiation slow down rate of momentum transfer.

The solution of the present work also validates with the previous result obtained by Seth et al.^47^, Asogwa et al.^48^, Seth et al.^49^ and Kataria and Patel^50^ in particular case.

The governing equations of the present problem are solved using Laplace transform technique. The problem is idealized by imposing some realistic constraints (e.g., viscous dissipation, Joule heating, effect of suction, induced magnetic field are neglected for mathematical simplicity). The same problem may be re- investigated by removing or reducing number of constraints. In this context, some numerical and computational techniques like Runge- Kutta method, shooting method, Crank- Nicolson method etc. may be suggested.

## Supplementary Information

Supplementary Information.

## Data Availability

All data generated or analysed during this study are included in this published article and its supplementary information files.

## List of symbols

$a$: Surface acceleration parameter
$\overrightarrow {B}$: Magnetic flux density
$B_{0}$: Strength of the applied magnetic field $\left(\frac{\mathrm{Weber}}{{\mathrm{m}}^{2}}\right)$
$C$: Molar species concentration $\left(\frac{\mathrm{mol}}{{\mathrm{m}}^{3}}\right)$
$C_{p}$: Specific heat at constant pressure $\left(\frac{\mathrm{J}}{\mathrm{kg K}}\right)$
$$C_{s}$$: Concentration susceptibility
$C_{\infty }$: Concentration far away from the plate $\left(\frac{\mathrm{mol}}{{\mathrm{m}}^{3}}\right)$
$C_{w}$: Concentration at the plate $\left(\frac{\mathrm{mol}}{{\mathrm{m}}^{3}}\right)$
$$D_{M}$$: Mass diffusivity $$\left(\frac{{\mathrm{m}}^{2}}{\mathrm{s}}\right)$$
$$Du$$: Dufour number
$\overrightarrow {g}$: Gravitation acceleration vector
$$g$$: Gravitational acceleration $$\left(\frac{\mathrm{m}}{{\mathrm{s}}^{2}}\right)$$
$Gr$: Thermal Grashof number
$Gm$: Solutal Grashof number
$K_{T}$: Thermal diffusion ratio
$$K*$$: Porosity parameter
$\overrightarrow {J}$: Current density vector $\left(\frac{\mathrm{A}}{{\mathrm{m}}^{2}}\right)$
$\overline{K}$: Chemical reaction rate $\left(\frac{\mathrm{mol}}{{\mathrm{m}}^{2}\mathrm{s}}\right)$
$K$: Chemical reaction parameter
$$M$$: Magnetic parameter
$$N$$: Radiation parameter
$$p$$: Pressure $$\left(\frac{\mathrm{N}}{{\mathrm{m}}^{2}}\right)$$
$$\Pr$$: Prandtl number
$\overrightarrow {q}$: Fluid velocity vector
$\overrightarrow {{q_{r} }}$: Radiation heat flux vector
$q_{r}$: Radiation heat flux $\left(\frac{\mathrm{W}}{{\mathrm{m}}^{2}}\right)$
$$Sc$$: Schmidt number
$$t^{\prime}$$: Time $$\left(\mathrm{s}\right)$$
$t_{{_{0} }}$: Critical time for rampedness $\left(\mathrm{s}\right)$
$t_{1}$: Non- dimensional critical time for rampedness
$$T$$: Fluid temperature $$\left(\mathrm{K}\right)$$
$T_{w}$: Temperature at the plate $\left(\mathrm{K}\right)$
$$T_{\infty }$$: Undisturbed temperature $$\left(\mathrm{K}\right)$$
$u^{\prime}$: X-component of fluid velocity $\left(\frac{\mathrm{m}}{\mathrm{s}}\right)$
$$U_{0}$$: Plate velocity $$\left(\frac{m}{s}\right)$$

## Greek symbols

$\mu$: Coefficient of viscosity $\left(\frac{\mathrm{kg}}{\mathrm{m s}}\right)$
$$\sigma$$: Electrical conductivity $$\left(\frac{\mathrm{S}}{\mathrm{m}}\right)$$
$\sigma^{*}$: Stefan-Boltzmann constant $\left(\frac{\mathrm{W}}{{\mathrm{m}}^{2} {\mathrm{K}}^{4}}\right)$
$$\rho$$: Fluid density $$\left(\frac{\mathrm{kg}}{{\mathrm{m}}^{3}}\right)$$
$\rho_{\infty }$: Fluid density far away from the plate $\left(\frac{\mathrm{kg}}{{\mathrm{m}}^{3}}\right)$
$$\kappa$$: Thermal conductivity $$\left(\frac{\mathrm{W}}{\mathrm{m K}}\right)$$
$\kappa^{*}$: Mean absorption constant $\left(\frac{1}{\mathrm{m}}\right)$
$\beta$: Volumetric coefficient of thermal expansion $\left(\frac{1}{\mathrm{K}}\right)$
$\overline{\beta }$: Volumetric coefficient of solutal expansion $\left(\frac{1}{\mathrm{K mol}}\right)$
$$\nu$$: Kinematic viscosity $$\left(\frac{{\mathrm{m}}^{2}}{\mathrm{s}}\right)$$

## Subscripts

$w$: Refers to physical quantity at the plate
$\infty$: Refers to physical quantity far away from the plate
