# Supplementary material for: Dufour effect on unsteady MHD flow past a vertical plate embedded in porous medium with ramped temperature
Source: Sci Rep. 2022 Aug 3;12:13343. doi: 10.1038/s41598-022-15603-x (PMC9349212; doi:10.1038/s41598-022-15603-x)
Supplement: Supplementary file 1 — Supplementary Information. [file 41598_2022_15603_MOESM1_ESM.docx]

**Appendix**

1. $\overline{f\left(x_{1},x_{2},x_{3},...,x_{n},t\right)}=f\left(x_{1},x_{2},x_{3},...,x_{n},t-t_{1}\right)H\left(t-t_{1}\right),$where $H\left(t-t_{1}\right)=\left\{\begin{matrix} 1,t>t_{1} \\ 0,t<t_{1} \end{matrix}\right.$

$$\Delta f=f-\bar{f}$$

$$\psi\left(\xi,\eta,y,t\right)=\frac{1}{2}\left[e^{\sqrt{\xi\eta}y}erfc\left(\frac{y}{2}\sqrt{\frac{\xi}{t}}+\sqrt{\eta t}\right)+e^{-\sqrt{\xi\eta}y}erfc\left(\frac{y}{2}\sqrt{\frac{\xi}{t}}-\sqrt{\eta t}\right)\right]$$

$$\lambda\left(\xi,y,t\right)=t\left[\left(1+\frac{y^{2}\xi}{2t}\right)erfc\left(\frac{\sqrt{\xi}y}{2\sqrt{t}}\right)-y\sqrt{\frac{\xi}{\pi t}}e^{-\frac{\xi y^{2}}{4t}}\right]$$

$$\Psi\left(\xi,\eta,\zeta,y,t\right)=e^{\zeta t}\psi\left(\xi,\eta+\zeta,y,t\right)$$

$$P\left(\xi,y,t\right)=\frac{\sqrt{\xi}y}{2\sqrt{\pi}t^{\frac{3}{2}}}e^{-\frac{\xi y^{2}}{4t}}$$

$$l\left(\xi,\eta,y,t\right)=\frac{\sqrt{\xi}ye^{-\eta t-\frac{\xi y^{2}}{4t}}}{2\sqrt{\pi}t^{\frac{3}{2}}}$$

$$h\left(\xi,y,t\right)=\frac{1}{2}\left[e^{\sqrt{\xi}y}erfc\left(\frac{y}{2\sqrt{t}}+\sqrt{\xi}t\right)+e^{-\sqrt{\xi}y}erfc\left(\frac{y}{2\sqrt{t}}-\sqrt{\xi}t\right)\right]$$

$$r\left(\xi,y,t\right)=\frac{1}{2}\left[\left(t+\frac{y}{2\sqrt{\xi}}\right)e^{\sqrt{\xi}y}erfc\left(\frac{y}{2\sqrt{t}}+\sqrt{\xi t}\right)+\left(t-\frac{y}{2\sqrt{\xi}}\right)e^{-\sqrt{\xi}y}erfc\left(\frac{y}{2\sqrt{t}}-\sqrt{\xi t}\right)\right]$$

$$q\left(\xi,y,t\right)=\frac{ye^{-\xi t-\frac{y^{2}}{4t}}}{2\sqrt{\pi}t^{\frac{3}{2}}}$$

$$\nu\left(\xi,t\right)=-2\sqrt{\frac{\xi t}{\pi}}$$

$$\alpha\left(\xi\right)=-\frac{2\xi}{\sqrt{\pi}}$$

$$\Omega\left(\xi,\eta,t\right)=-\left[\sqrt{\frac{\xi}{\pi t}}e^{-\eta t}+\sqrt{\xi\eta}.erf\left(\sqrt{\eta t}\right)\right]$$

$$Z\left(\xi,\eta,\zeta,t\right)=e^{\zeta t}\Omega\left(\xi,\eta+\zeta,t\right)$$

$$I\left(\xi,t\right)=\frac{\xi}{2\sqrt{\pi}t^{\frac{3}{2}}}$$

$$T\left(\xi,\eta,t\right)=\frac{\sqrt{\xi}e^{-\eta t}}{2\sqrt{\pi}t^{\frac{3}{2}}}$$

$$N\left(\xi,t\right)=-\left[\sqrt{\xi}erf\left(\sqrt{\xi t}\right)+\frac{1}{\sqrt{\pi t}}e^{-\xi t}\right]$$

$$O\left(\xi,t\right)=-\left[\left(\frac{2}{\sqrt{\xi}}+\sqrt{\xi t}\right)erf\left(\sqrt{\xi t}\right)+\sqrt{\frac{t}{\pi}}e^{-\xi t}\right]$$

$$Y\left(\xi,t\right)=\frac{e^{-\xi t}}{2\sqrt{\pi}t^{\frac{3}{2}}}$$
